# Field‐Driven Proton Transport in Water Revealed by Machine Learning Molecular Dynamics

**DOI:** 10.1002/advs.78513

**Published:** 2026-10-29

**Authors:** Alicia van Hees, Zhan‐Yun Zhang, Linnéa Andersson, Chao Zhang

**Affiliations:** ^1^ Department of Chemistry‐Ångström Laboratory Uppsala University Uppsala Sweden; ^2^ Wallenberg Initiative Materials Science for Sustainability Uppsala University Uppsala Sweden

**Keywords:** electrolyte solution, ion transport, machine learning, non‐equilibrium molecular dynamics, proton transfer

## Abstract

Excess‐proton transport in water arises from the coupling of proton transfer (PT), hydrogen‐bond rearrangement, and solvent polarization. While equilibrium Grotthuss diffusion has been widely studied, its response under strong electric fields remains difficult to resolve because simulations must capture both chemical reactivity and induced polarization over long time scales. Here, we have developed a reactive and polarizable machine‐learning interatomic potential for molecular dynamics simulations of the hydrated excess proton in water. A fine‐tuned PiNet2 model provides an accurate zero‐field reference for structure and diffusion, while an AD(OS)‐based finite‐field coupling enables non‐equilibrium simulations under an applied electric field. In the linear‐response regime, below 0.04 V/Å, the proton mobility obtained from drift velocity agrees closely with the equilibrium diffusion coefficient. The proton drift velocity remains nearly linear with field strength up to 0.15 V/Å, a field strength at which the ionic mobility of a constrained hydronium ion decreases. Our simulations revealed a distinct field‐response of protonated water, whose hydrogen‐bond network largely preserves its structural fluctuations and mobility without a comparable dielectric‐saturation signature. Furthermore, free‐energy analysis shows that strong fields stabilize the hydronium species with four‐fold H‐bond coordination.

## Graphical abstract

Using a reactive and polarizable machine‐learning molecular dynamics (MLMD) framework, the authors show that excess‐proton drift remains nearly linear with applied electric field up to 0.15 V/Å. It is also found that the protonic defect mitigates the field‐induced structural changes observed in neat water, revealing a distinct high‐field response of protonated water.

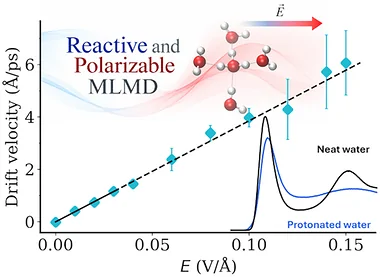

## 1 Introduction

Proton transport in liquid water is a prototypical example of molecular charge migration in a fluctuating hydrogen‐bond network. Unlike ordinary ionic diffusion, the excess proton does not move only through the center‐of‐mass motion of a tagged hydronium ion. Instead, charge migration is dominated by structural diffusion, in which covalent and hydrogen bonds reorganize so that the protonic defect is transferred between neighboring water molecules. This mechanism, historically associated with Grotthuss transport, has been refined through the Eigen and Zundel pictures of proton solvation, modern spectroscopy, and molecular simulation [1, 2, 3, 4, 5]. The central challenge is that proton mobility is controlled by a coupled process: local proton sharing, hydrogen‐bond rearrangement, solvent polarization, and nuclear quantum fluctuations all occur on comparable time scales.

Atomistic simulation has played a crucial role in resolving this enigma. Early ab initio molecular dynamics (AIMD) studies established that the hydrated excess proton is not a static chemical species, but a dynamic defect whose identity alternates between Eigen‐like and Zundel‐like motifs [6, 7, 8]. Subsequent first‐principles and empirical valence bond simulations emphasized the importance of hydrogen‐bond topology, presolvation, and correlated proton hops in determining the observed mobility [9, 10, 11, 12]. More recently, AIMD with improved exchange‐correlation treatments has clarified the asymmetry between hydronium and hydroxide diffusion: while hydronium transfer can proceed through temporally correlated structural diffusion, hydroxide is slowed by a more stable hypercoordinated solvation structure that suppresses correlated PT [13]. These studies collectively show that quantitative proton transport cannot be understood from static solvation structure alone; it requires a dynamical description of bond rearrangement in the surrounding water network. Recent neural‐network MD simulation has further sharpened this picture by extending the accessible time and length scales while retaining near‐first‐principles accuracy. Gomez, Thompson and Laage used neural‐network potentials together with nuclear quantum dynamics [14] to show that aqueous PT proceeds through a three‐step sequence controlled by two successive hydrogen‐bond exchange events: one prepares the acceptor environment for PT, while the second stabilizes the new proton donor environment and suppresses rapid back‐transfer. It also highlights that a realistic model must describe both chemical reactivity and the dielectric response of water, since the excess proton is inseparable from the fluctuating polarization field of the solvent.

A further complication arises under an external electric field or applied electrochemical bias. In electrochemical systems, proton transport commonly occurs in the presence of interfacial fields, potential drops, confinement, and nonequilibrium driving forces. Voth and co‐workers studied proton transport through an electrolyte layer between platinum electrodes under applied voltage using reactive MD simulations and decomposed the transport into vehicular and Grotthuss hopping components [15]. They found that increasing the applied voltage enhances the hopping contribution and changes the correlation between vehicular and structural motion. This result suggests that PT under bias does not happen in the same way as the classical Grotthuss diffusion. Under strong fields, ordinary ionic mobility is expected to decrease because the orientational polarization of water saturates, increasing dielectric friction [16]. Whether the same logic applies to the hydrated excess proton is unclear, because proton charge migration is partly structural rather than purely vehicular. However, direct first‐principles simulations of such non‐equilibrium proton dynamics remain limited by computational cost, while many reactive force‐field or empirical valence‐bond approaches require system‐specific parametrization and may not fully capture electronic polarization.

Here we address this challenge by combining a reactive machine‐learning interatomic potential (MLIP) with a learned description of the polarization and Born effective charge. Our approach builds on the PiNNAcLe‐FT workflow for foundational model distillation and adaptive learn‐on‐the‐fly model fine‐tuning with new DFT labels. In the present work, we fine‐tune a PiNet2 potential for the excess proton in liquid water and augment it with a polarization and Born‐effective‐charge model AD(OS) trained from finite‐field DFT calculations. This combination enables machine learning molecular dynamics (MLMD) simulations in which PT, water reorganization, and coupling to an applied electric field are treated within a unified framework (Figure 1).

**FIGURE 1:**
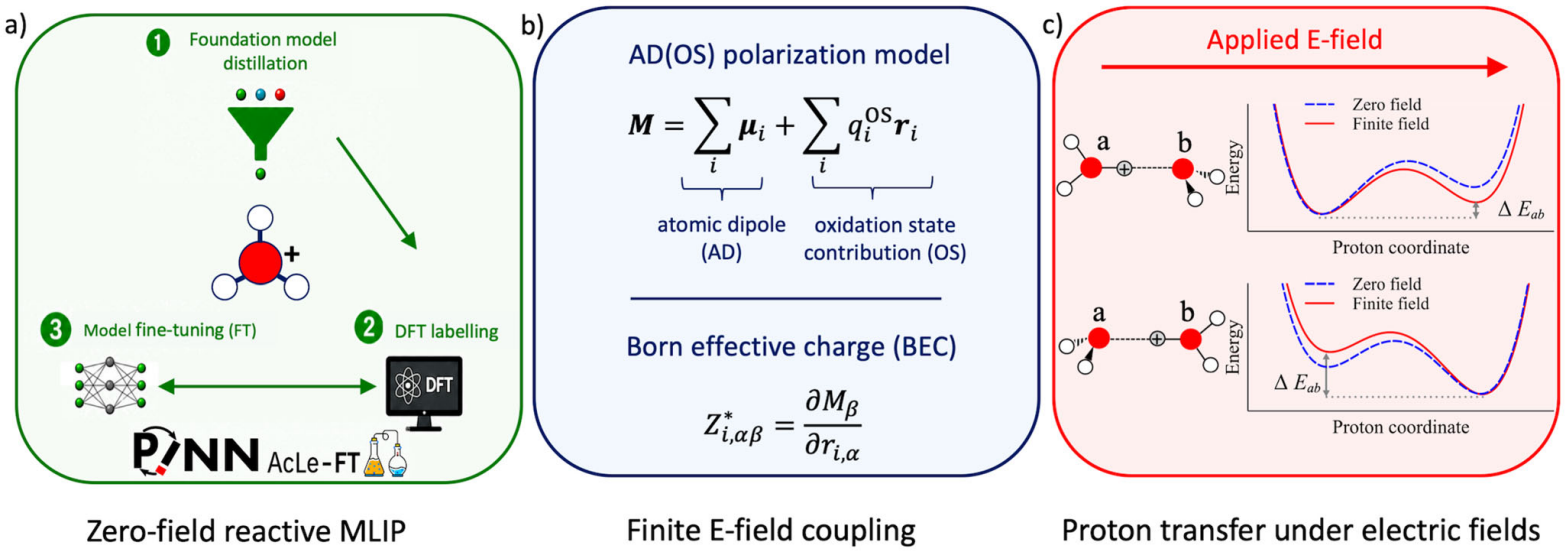
Conceptual workflow of reactive and polarizable MLMD simulations of excess‐proton dynamics in water. (a) The zero‐field reactive MLIP is constructed by PiNNAcLe‐FT through foundation‐model distillation, DFT labelling, and model fine‐tuning for the protonated water system. (b) The AD(OS) model then introduces finite‐field coupling through a consistent polarization – Born effective charge description, enabling NEMD simulations of proton drift under an applied electric field. (c) In the strong‐field regime, the applied field modifies significantly the PT energy profiles. $\Delta E_{ab}=E_{a}-E_{b}$ is the energy gap for a vertical transition between two proton states where the excess proton is either attached to the left oxygen ($O_{a}$) or the right oxygen ($O_{b}$), where the rest of nuclear coordinates are fixed.

In the following, we first validate the resulting reactive and polarizable model at equilibrium by comparing the structure and diffusion of water and the excess proton with experimental and first‐principles benchmarks. We then perform finite‐field simulations and obtain the proton mobility from the field‐induced drift velocity, providing an independent non‐equilibrium estimate of the diffusion coefficient through the Einstein relation. Finally, we use the model to analyze how the protonic defect and the surrounding solvent respond beyond the strictly equilibrium picture, with particular attention to the relative roles of PT energetics, hydrogen‐bond coordination, and solvent polarization under a higher field strength. By doing so, we establish a practical route for nanosecond‐scale simulations of aqueous proton transport in which chemical reactivity and dielectric response are treated consistently.

## 2 Results and Discussion

### 2.1 Reactive MLIP for the Protonated Water System

Efficient and high‐fidelity MLIPs are essential for studying proton transport in liquid water over extended timescales. For the efficiency, we have applied the foundation model distillation workflow PiNNAcLe [17, 18]. As shown in our previous work [18], although the distilled bespoke model can reproduce the foundation model with comparable accuracy, the foundation model itself may still deviate noticeably from DFT reference data for a given system, even at the same level of theory. Therefore, to achieve high fidelity, in this work, we introduced a fine‐turning step in the PiNNAcLe‐FT workflow. In a nutshell, PiNNAcLe‐FT distilled the MACE‐MATPES‐r2SCAN‐0 foundation model [19, 20] into our lightweight PiNet2 architecture [21] and then performed an automated fine‐tuning of PiNet2 model [21] at the $r^{2}\mathrm{SCAN}$ level of theory [22] with fully conveged DFT labels (see Figure S2) obtained from CP2K [23]. This levitates the PiNet2‐prior model that matches the foundation model accuracy (See Figures S3 and S4) to the PiNet2‐ft model that agrees with the DFT accuracy. Through the fine‐tuning with PiNNAcLe runs (see Figure S5), the validation errors of the PiNet2‐ft models are below 1 meV/atom for energy per atom and 31 meV/Å for forces. Detailed description of the workflow can be found in of the Supporting Information (Sections S3 and S4 therein).

Based on the production run of the PiNet2‐ft at 330 K, we analyzed the radial distribution functions (RDFs), g(r), for species of interest. Figure 2a presents the results between oxygen atoms of water molecules, together with the reference from an AIMD trajectory at the $r^{2}\mathrm{SCAN}$/TZV2P level (See Section S1 and Figure S1). As one can see, the first peak of the oxygen‐oxygen RDF predicted by the PiNet2‐prior models is noticeably higher than that of the DFT reference, indicating an overstructured liquid‐water network inherited from the foundation model. In contrast, the PiNet2‐ft models reproduce the reference RDFs faithfully. Figure 2b–d illustrates the RDFs between atomic species in hydronium ions (denoted by superscript *) and those in water molecules, where a similar improvement was also seen there.

**FIGURE 2:**
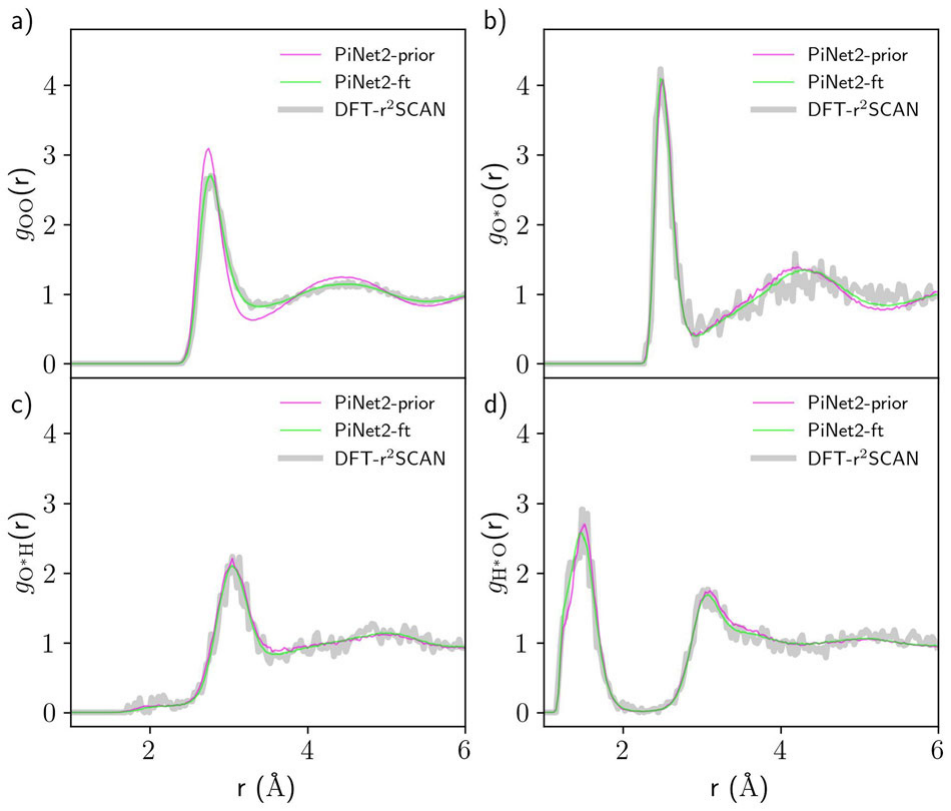
Radial distribution functions (RDF) g(r) of selected species at 330 K. Superscripts 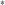 identify atoms in the hydronium ion, while other atoms are associated with water molecules. (a) The RDF between O atoms and O atoms; (b) The RDF of O atoms around $O^{*}$ atoms; (c) The RDF of H atoms around $O^{*}$ atoms; (d) The RDF of O atoms around $H^{*}$ atoms. The standard deviations of PiNet2‐prior and PiNet2‐ft models were estimated from three independent 5 ns NVT trajectories, and shown in transparent colors. The DFT results were obtained from a 15 ps NVT simulation at the matched $r^{2}\mathrm{SCAN}$/TZV2P level of theory.

### 2.2 Proton Dynamics at Equilibrium

The dynamics properties were further analyzed using the same trajectories as those used for the RDF analysis. The extracted mean squared displacements (MSDs) of all oxygen atoms (including hydronium oxygen atoms) and the proton are shown in Figures S6 and S7, respectively. On one hand, the PiNet2‐prior models at 330K yield a mean $D_{o}$ of $1.05\times 10^{-9}\;m^{2}$/s, approximately half of the experimental value ($2.3\times 10^{-9}\;m^{2}$/s), whereas the fine‐tuned models give 2.4 $\times 10^{-9}\;m^{2}$/s, in much closer agreement with experiment. On the other hand, the PiNet2‐prior and PiNet2‐ft models reported comparable values for $D_{H^{+}}$ when their uncertainties are taken into account. Regarding the ratio of $D_{o}$/$D_{H^{+}}$, the value from PiNet2‐ft models is 0.20, which is smaller than that from the experiment (0.245) but very comparable to the value of 0.207 reported from MLIP‐based thermostatted ring polymer simulations at the revPBE0‐D3 level and 300 K [14]. Therefore, the 30 K temperature increase above room temperature used to mimic nuclear quantum effects in liquid water simulation [24] turned out to be effective for dynamical properties (Table 1).

**TABLE 1:** Self‐diffusion coefficients of oxygen atoms ($D_{O}$) and proton ($D_{H^{+}}$) as well as their ratio $D_{O}/D_{H^{+}}$. EMD: equilibrium MD and NEMD: non‐equilibrium MD.

Condition	$D_{O}$ (10 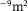 /s)	$D_{H^{+}}$ (10 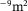 /s)	$D_{O}/D_{H^{+}}$
Exp. (298K)	2.30 [25]	9.4 [26]	0.24
PiNet2‐ft (EMD, 300K)	1.0 ± 0.2	6.9 ± 1.0	0.14 ± 0.01
PiNet2‐ft (EMD, 330K)	2.4 ± 0.2	11.7 ± 0.6	0.20 ± 0.03
PiNet2‐ft (NEMD, 330K)	N/A	11.2 ± 0.4^1^	N/A

1 Note the excellent agreement between the EMD and NEMD estimations of the proton diffusion constant, which cross‐validates the framework of reactive and polarizable MLMD simulations.

### 2.3 Proton Migration Under a Finite Electric Field

After setting the stage, now we can look in to the finite‐field MLMD simulations. Our implementation (see Section S6) was inspired by the perturbed neural network potential [27]. The key difference is that we have employed the atomic dipole plus oxidation state AD(OS) model [21] to describe the total dipole moment and the Born effective charge consistently, which satisfies the modern theory of polarization [28, 29] and is essential for modeling electrolyte solutions (see Figures S8 and S9). Then by monitoring the displacement of the total dipole moment $\Delta M_{z}$ over the time period Δt in the direction of the applied electric field $E_{z}$, one can extract the mobility coefficients of the excess proton:

(1) $$\mu_{H^{+},\;z}=\frac{{\bar{v}}_{H^{+},\;z}}{E_{z}}=\frac{\Delta M_{z}}{qE_{z}\Delta t}$$

where ${\bar{v}}_{H^{+},\;z}$ is the proton drift velocity and q is the nominal charge of the excess proton. The long‐time linear growth of $\Delta M_{z}$ reflects net charge transport, i.e., the proton drift velocity, whereas bounded orientational polarization therein from water contributes only to transient response [30]. Then, the self‐diffusion coefficient can be readily obtained from the Einstein relation in kinetic theory $D_{H^{+}}=\mu_{H^{+}}k_{B}T/q$.

As showed in Figure S10, the convergence of the drift velocity $v_{H}^{+}$ was reached within 500 ps. By fitting the resulting drift velocities to the applied electric field (up to 0.03 V/Å) with a linear function and converting it into the self‐diffusion coefficient $D_{H^{+}}$, we obtained a value of $11.2\times 10^{-9}m^{2}$/s (Table 1), which comes in excellent agreement with that obtained from the MSD using the equilibrium simulations. This is a direct proof that the reactive and polarizable MLMD scheme shown in Figure 1 works.

When inspecting the dependence of the proton drift velocity on the field strength (Figure 3a), we observe a linearity between the proton drift velocity and the applied electric field that holds up to 0.15 V/Å. To distinguish whether this observation is unique to the excess proton, which involves both vehicular and structural diffusion, we also carried out a control study where all O–H bond in the system were constrained and no proton transfer event is allowed to happen in the simulation. The outcome of this control study is shown in (Figure 3b). Clearly, the classical hydronium ion diffuses much slower as compared to the free proton. More importantly, the drift velocities of classical hydronium ion become saturated with the increase of the field strength. This trend comes in in agreement with the results from classical force field simulations of monovalent alkaline ions, e.g., ${\mathrm{Na}}^{+}$, which are attributed to the dielectric saturation [16]. Despite of this qualitative difference shown in Figure 3, it should be pointed out that there are large error bars associated with the proton drift velocities obtained at high field, similar to what was observed previously for monovalent alkaline ions from perturbed neural network potential‐based simulations [31]. In addition, the system size and the (effective) proton concentration can also influence the results (Figure S11). Therefore, in the following, we shift our attention to understand the how water structure responses differently at high field and how these responses show up differently in the presence of excess proton in the system.

**FIGURE 3:**
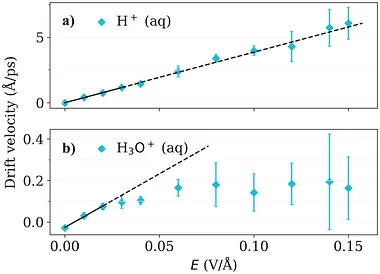
(a) Drift velocity ${\bar{v}}_{H^{+}}$ as a function of the applied electric field E. (b) The corresponding plot for the case where all O–H bonds were constrained, so no proton transfer can happen. The solid line represents a linear fit to the data up to E=0.03 V/Å, and the dashed extension is included as a guide to the eye. No field‐included water dissociation was observed in any MLMD simulations reported here.

### 2.4 Structural Responses of Neat Water and Protonic Water Under a Finite Electric Field

Water, as a dielectric, polarize strongly under an applied electric field. Indeed, for the neat water, we see a rapid decrement of the static dielectric constant (computed from $\varepsilon =\varepsilon_{\infty }+4\pi \left\langle M_{z}\right\rangle /\left(E_{z}\Omega \right)$ [32]) to below 30 at E=0.15 V/Å (see Figure 4a). It is accompanied by a significant structuring of the water structure (see Figure 4c and the tetrahedral order parameter [33] shown in Figure S13b), a lowering of the water self‐diffusion coefficients (Figure 4b) as well as an increase of H‐bond per water molecule (Figure S14). These results confirm what were reported in the previous work on the electrofreezing of liquid water at ambient conditions [34].

**FIGURE 4:**
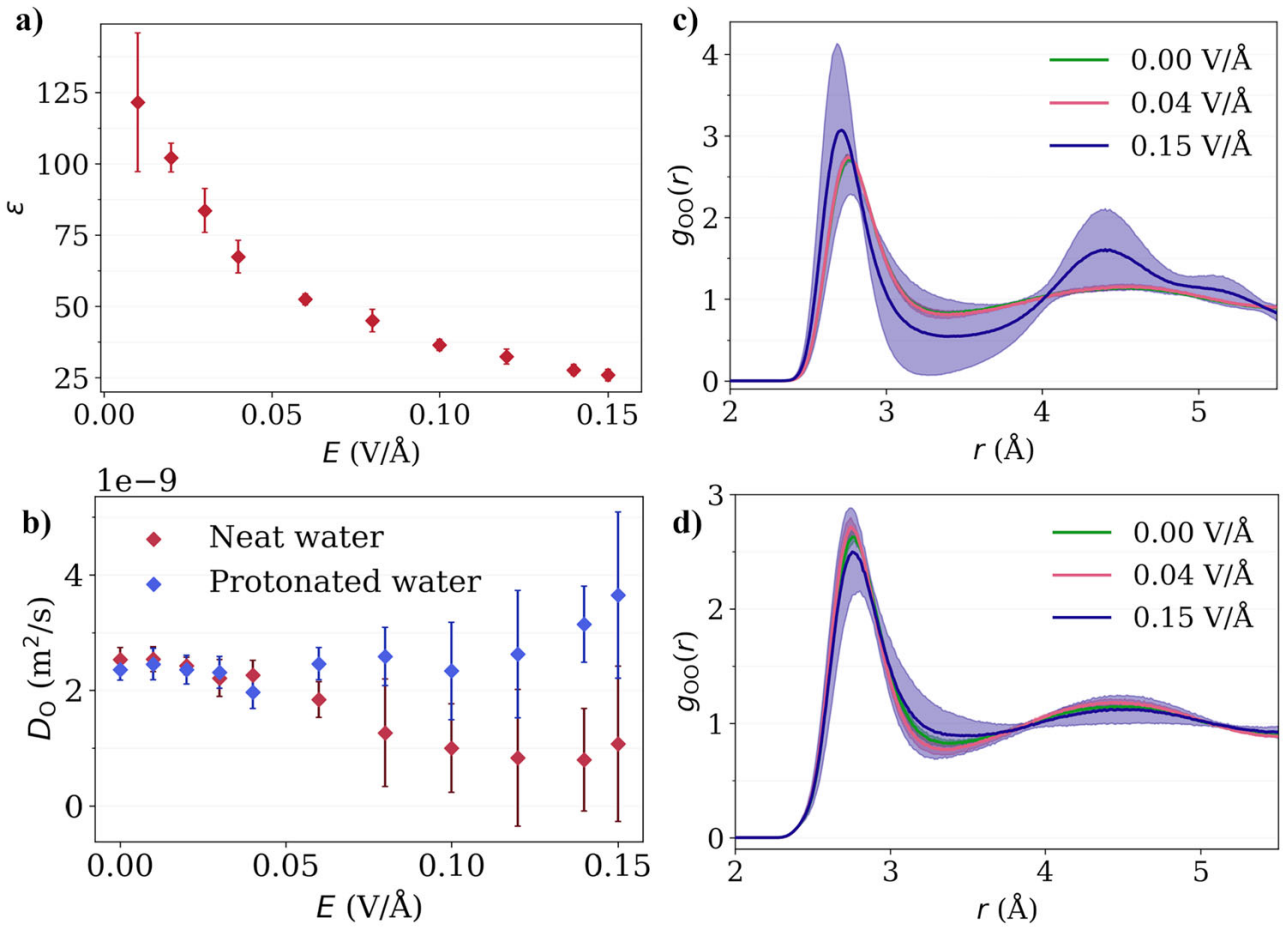
The effect of electric fields on water dielectric constant, diffusion and structure. (a) The decrease of the static dielectric constant ε of neat water with increasing field strength. (b) The field‐dependent self‐diffusion coefficient of oxygen atoms in neat water and in protonated water. (c) and (d) O–O radial distribution functions $g_{\mathrm{OO}}$ in neat water (top) and protonated water (bottom) at E=0.00 V/Å, E=0.04 V/Å, and E=0.15 V/Å. The shaded regions denote one standard deviation.

However, the situation is quite different for the protonated water system. The oxygen–oxygen RDFs only changes mildly under a strong field as compared to the ones at lower and zero field (Figure 4d), so does the tetrahedral order parameter (Figure S13a). Then, the water self‐diffusion coefficients keep basically unchanged (or even slightly enhanced) as shown in Figure 4b as well as in Figure S12 for a larger simulation system. This correlates well with a slightly decrease in the number of H‐bonds per water molecule in the protonated water system when the field strength increases (Figure S14). This echos the same observation reported previously using AIMD simulations [35]. In addition, it is found that water self‐diffusion coefficients appear to be consistently faster along the direction of applied field than their counterparts in the perpendicular direction (Figure S15). Similar observation was made previously in classical force field‐based MD simulations [36].

Despite of these interesting findings, it does not escape our eyes that there are large structural variations found in both neat water and protonated water systems (Figure 4c,d as well as Figure S16). We suspect that this is the origin of the large error bars associated with transport properties from our simulations (Figures 4b and 3). Given that this type of reactive and polarizable MLMD simulations that we present in this work may get popular in the near future, it would be necessary to understand what contributes to this observed large structural heterogeneity at high field. As discussed in the Sections S11–S13, the MLIP prediction accuracies for forces, Born effective charge and polarization remain excellent at high field and no notable difference in error metrics was observed when taking trajectory snapshots from structurally distinct systems under the same high field condition. Therefore, our conclusion is that there are long‐lived metastable states in both neat water and protonated water systems at high field. This point is definitely worth further investigations but it goes beyond the scope of this work.

Besides structural responses of neat water and protonic water presented so far, it would be also interesting to see how the local structure changes around the excess proton under a finite electric field. For this purpose, we have applied the analysis of the free energy surface of PT developed previously [14], where the two reaction coordinates are the hydrogen‐bond coordination numbers $\left(n_{a},n_{b}\right)$ of the oxygen atoms involved in the PT as well as the vertical energy gap $\Delta E_{ab}$ between two PT states (also see Figure 1c). It is worth noting that a and b here refer to the “left” and “right” oxygen along the PT coordinate (in the z direction). Therefore, for the forward reaction ($\Delta E_{ab}<0$), “left” oxygen is the proton donor and the “right” oxygen is the proton accepter; their identities get swapped for the backward reaction ($\Delta E_{ab}>0$).

As shown in Figure 5a, we can recover the same FES in the zero‐field MLMD simulations as reported previously [14] despite of not considering the nuclear quantum effect directly. Then, it is found that the FES does not change qualitatively when the applied electric field is smaller than E=0.04 V/Å (Figure 5b). This is within the expected linear‐response regime. However, at a high field strength of E=0.15 V/Å (Figure 5c), (3a,4b) minimum becomes much more populated as compared to (4a,3b) minimum because the proton migrates along the direction of the applied E‐field. In such case, the forward PT reaction from $O_{a}$ to $O_{b}$ is more favored as compared to the backward PT reaction from $O_{b}$ to $O_{a}$. Furthermore, agreed with intuition, the field‐coupling term $-E_{z}\Delta M_{ab,z}$ destabilizes the state where the excess proton is attached to the left oxygen ($O_{a}$) and stabilize the state where the excess proton is attached to the right oxygen ($O_{b}$) (Figures 1c and 5f).

**FIGURE 5:**
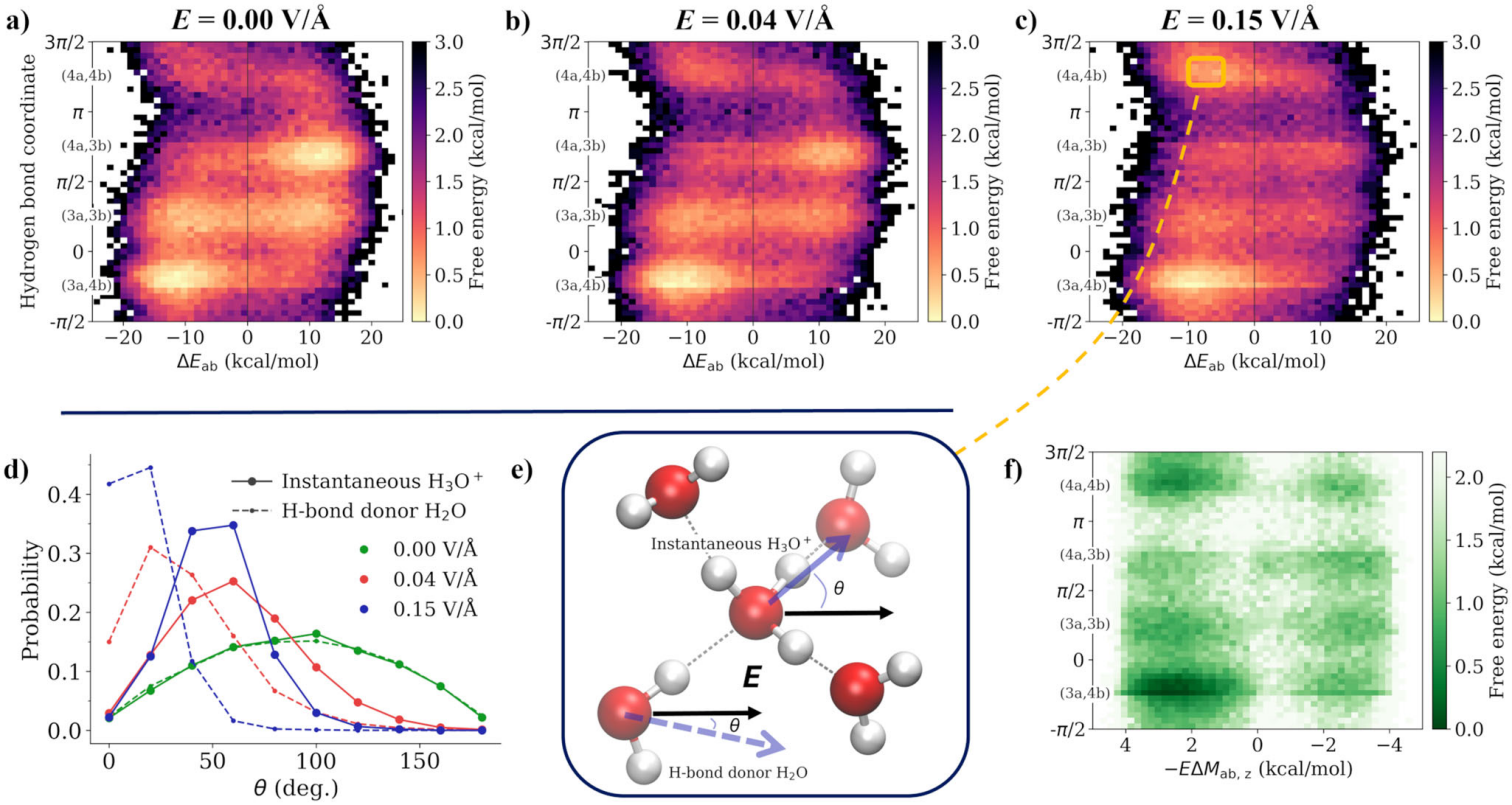
(a)– (c) Free energy surfaces (FESs) of PT as a function of the hydrogen‐bond coordinate and the energy gap $\Delta E_{ab}$ in the PT at E=0.0 V/Å, E=0.04 V/Å and E=0.15 V/Å respectively. The *y*‐axes represent the ”hydrogen bond coordinate”, which is described in detail in Ref. [14]. In summary, a label such as (4a, 3b) denotes that the assigned proton donor ”a” partakes in four hydrogen bonds and the assigned proton acceptor ”b” partakes in three hydrogen bonds. (d) The angles that the instantaneous hydronium species and its hydrogen bond donor make with the electric field direction. For an instantaneous hydronium species, its directional vector is defined as the normal to the plane composed by its three hydrogen atoms. The normal in the direction pointing away from the oxygen atom is always chosen. For a water molecule, the directional vector is instead the bisector of the H–O–H angle. In both cases, the angle θ is the angle between this directional vector and the positive z‐direction in which the electric field is applied. (e) The molecular structure of a four‐fold coordinated hydronium species contributing to the (4a,4b) minimum on the FES at E=0.15 V/Å. (f) FES of proton transfer as a function of the hydrogen‐bond coordinate and the field‐dependent contribution to the energy gap, i.e., $-E\Delta M_{ab,z}$, at E=0.15 V/Å.

What is really interesting is that the metastable (4a,4b) state at zero field becomes stable at a higher applied field (Figure 5c,e). Because the hydronium ion is known as an amphiphilic ion, the four‐fold hydrogen‐bond coordination is expected to be unfavorable in water [37, 38, 39]. It is found that both the hydronium ion and the water molecule acting as the H‐bond donor align their dipoles with the applied field (Figures 5d), and this helps to populate the four‐fold coordinate of the hydronium ion. This is further supported by that the first peak of the intermolecular RDF between H atoms and the hydronium O becomes much more pronounced at E=0.15 V/Å as compared to those at E=0.04 V/Å and E=0.00 V/Å (Figure S22a) and the solid angle formed by the three hydrogen atoms in the hydronium ion becomes much smaller at E=0.15 V/Å (See Figure S22b).

## 3 Conclusion

We have developed a reactive and polarizable MLMD framework for simulating the excess proton in water under both equilibrium and finite‐field conditions. By combining a fine‐tuned PiNet2 MLIP with an AD(OS)‐based polarization and Born‐effective‐charge model, the present approach captures PT, hydrogen‐bond reorganization, and coupling to an external electric field within a single dynamical description. At equilibrium, the fine‐tuned model corrects the overstructured water network inherited from the foundation model and reproduces proton and water diffusion at a level consistent with first‐principles and experimental benchmarks. Under weak applied fields, the proton mobility obtained from the drift velocity agrees quantitatively with the corresponding diffusion coefficient from equilibrium molecular dynamics.

Our MLMD simulations showed that proton drift velocities remain nearly linear with the applied field up to 0.15 V/Å, a field strength at which the ionic mobility of a constrained hydronium ion decreases due to dielectric saturation. Nevertheless, significant structural heterogeneity is found in water structures at high field due to the existence of long‐lived metastable states, which makes the quantitative assessment of ion drift velocity more challenging with increased field strength. Our analysis of this structural heterogeneity at high field remains preliminary, and further investigations are needed to confirm and clarify this behavior, which may also occur in other aqueous electrolyte systems.

Our work revealed that the structural responses of water in protonated water systems are much less pronounced compared to those in neat water systems, in accord with previous findings from AIMD simulations [35]. Furthermore, we found that the applied field reshapes the proton‐transfer free‐energy surface: high‐field conditions stabilize additional hydrogen‐bond configurations, including a four‐fold coordinated proton‐transfer environment.

Together, these results establish reactive and polarizable MLMD as a practical route to studying proton transport under a finite electric field. They show that the interplay between electric fields, water structure and the excess proton leads to a rich landscape for structural, dynamical and dielectric properties. Therefore, both the computational framework developed here and the high‐field proton transport identified in the protonated water system are relevant to field‐driven ion transport in (aqueous) electrolyte solution, water dissociation [40], the electric double layer at electrode/electrolyte interfaces [41, 42], water‐air interfaces [43] as well as confined water, membranes, and other environments where electric fields impact molecular processes [44, 45].

## 4 Methods

The description of computational methods can be found in the Supporting Information, which includes computational setups for AIMD and MLMD simulations, comparison between AIMD simulation in this work and the literature, self‐consistent field (SCF) convergence in the DFT labels, construction of the reactive MLIP for the protonated water system, water and proton self‐diffusion coefficients from PiNet2‐ft models, finite‐field MLMD with AD(OS) models, convergence plots of proton drift velocity in finite field MLMD, finite size effect on the dynamical properties, H‐bond analyses and tetrahedral order parameters, anisotropic analyses of water diffusion, structural heterogenity at high field, accuracy assessments of MLIP at high field, polarization convergence and relaxation in neat water, and structural analysis of $H_{3}O^{+}$(aq) in response to applied E‐fields.

## Author Contributions

Alicia van Hees: writing – original draft, writing – review & editing, validation, investigation, formal analysis, data curation. Zhan‐Yun Zhang: writing – original draft, writing – review & editing, validation, investigation, formal analysis, data curation, software. Linnéa Andersson: writing – review & editing, methodology, software, investigation. Chao Zhang: writing – original Ddraft, writing – review & editing, investigation, supervision, resources, funding acquisition, methodology, conceptualization.

## Conflicts of Interest

The authors declare no conflicts of interest.

## Supporting information


**Supporting File**: advs78513‐sup‐0001‐SuppMat.pdf.

## Data Availability

A part of data that support the the findings of this study are available in GitHub at https://github.com/Teoroo‐CMC/PiNNAcLe‐FT. Further data that support the findings of this study are available from the corresponding author upon reasonable request.
