# Supplementary material for: Field‐Driven Proton Transport in Water Revealed by Machine Learning Molecular Dynamics
Source: Adv Sci (Weinh). 2026 Oct 29:e78513. Online ahead of print. doi: 10.1002/advs.78513 (PMC13720274; doi:10.1002/advs.78513)
Supplement: Supplementary file 1 [file ADVS-9999-e78513-s001.pdf]

Supporting Information: Field-Driven Proton Transport in Water Revealed by Machine Learning Molecular Dynamics

Alicia van Hees,[†,¶] Zhan-Yun Zhang,[†,¶] Linnéa Andersson,[†] and Chao Zhang[*,†,‡]

†*Department of Chemistry-Ångström Laboratory, Uppsala University, Lägerhyddsvägen 1, P. O. Box 538, 75121 Uppsala, Sweden*

‡*Wallenberg Initiative Materials Science for Sustainability, Uppsala University, 75121 Uppsala, Sweden*

¶*Contributed equally to this work*

E-mail: chao.zhang@kemi.uu.se

Contents

1 Computational setups for AIMD and MLMD simulations **S-4**

2 Comparison between AIMD simulation in this work and the literature **S-5**

3 SCF convergence in the DFT labels **S-6**

4 Construction of the reactive MLIP for the protonated water system **S-7**

4.1 Foundation model distillation S-7

4.2 Fine-tuning with PiNNAcLe-FT S-10

5 Water and proton self-diffusion coefficients from PiNet2-ft models **S-12**

6 Finite-field MLMD with AD(OS) models **S-15**

6.1 Finite field perturbation formula S-15

6.2 AD(OS) model for dipole and BEC predictions S-15

6.3 Dataset generation for the protonated water system S-16

6.4 Model training on the protonated water dataset S-16

7 Convergence plots of proton drift velocity in finite-field MLMD **S-19**

8 Finite size effect on the dynamical properties **S-20**

9 H-bond analyses and tetrahedral order parameters **S-22**

10 Anisotropic analyses of water diffusion **S-24**

11 Structural heterogeneity at high field **S-25**

12 Accuracy assessments of MLIP at high field **S-26**

13 Polarization convergence and relaxation in neat water **S-30**

14 Structural analysis of H_3O^+(aq) in response to applied E-fields S-32

References S-33

1 Computational setups for AIMD and MLMD simulations

DFT calculations were performed using the QUICKSTEP module in the CP2K package.[S1,S2] The r^2SCAN functional[S3] was used, and the kinetic energy cutoff for plane-wave expansion of the density was set to 1200 Ry. Core electrons were treated using Goedecker-Teter-Hutter (GTH) pseudopotentials,[S4] while the valence electron molecular orbitals were described using molecular optimized (MOLOPT) TZV2P basis sets.[S5] The self-consistent field (SCF) convergence criterion was set to 10^{-6}.

For the system, we used 63 water molecules and a hydronium ion in a periodic cubic cell with a length of 12.432 Å. MD simulations were carried out in the NVT ensemble at 330 K. The 30 K increase above room temperature is used to mimic the nuclear quantum effects in liquid water systems.[S6,S7] The integration time step was set as 0.5 fs. For MLIP-based MD simulations (MLMD for short), the Bussi-Donadio-Parrinello thermostat[S8] was employed, as implemented in the Atomic Simulation Environment (ASE) package.[S9] For DFTMD simulations, its implementation in CP2K was applied.

2 Comparison between AIMD simulation in this work and the literature

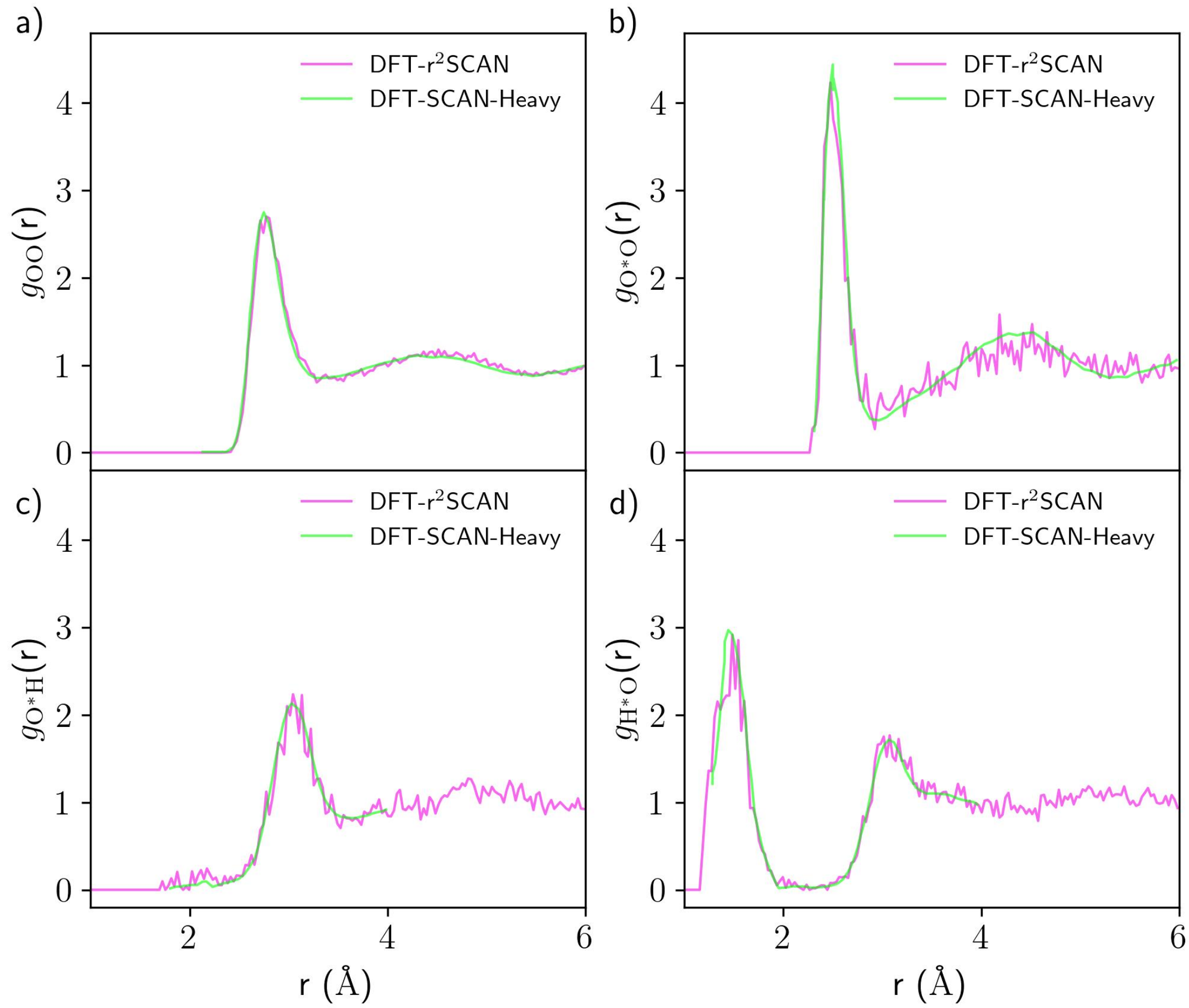

Figure S1: Radial distribution functions $g(r)$ of selected species from 15 ps DFTMD simulations at 330 K. Superscripts * identify atoms in the hydronium ion, while other atoms are associated with water molecules. (a) Distribution of O atoms around O atoms; (b) Distribution of O*atoms around O atoms; (c) Distribution of O* atoms around H atoms; (d) Distribution of H* atoms around O atoms. The standard deviations of PiNet2-prior models are shown in transparent colors. The DFT-SCAN-Heavy result in panel a was taken from Ref.,[S6] whereas those in panels bd were obtained from Ref.[S10] All reference DFT-SCAN-Heavy data correspond to heavy-water (D_2O) systems, i.e. deuterated water.

3 SCF convergence in the DFT labels

As recently pointed out,[S11] the total net force from the DFT labels should be accurate enough for an effective training of MLIP. The figure below shows the distribution of the total force for a number of snapshots extracted from the AIMD trajectories. The same SCF convergence criteria as listed in Section 1 was used in the subsequent fine-tuning with PiNNAcLe-FT.

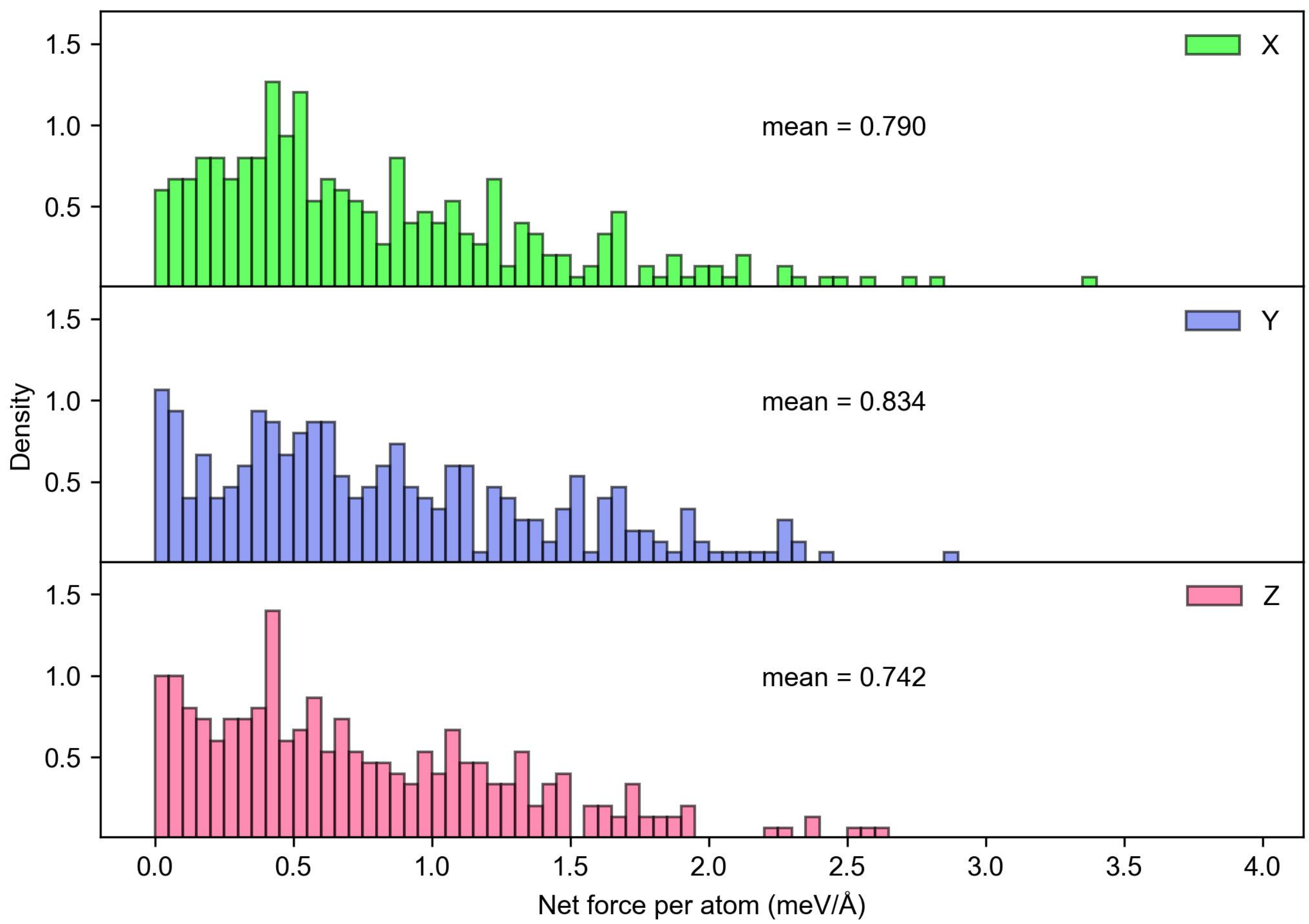

Figure S2: Distribution of net forces per atom along three directions obtained from 300 snapshots of the 15 ps AIMD trajectory at 330 K (snapshot interval: 50 fs).

4 Construction of the reactive MLIP for the protonated water system

4.1 Foundation model distillation

We performed knowledge distillation of the MACE-MATPES-r2SCAN-0 foundation model into our lightweight PiNet2 architecture. First, using the MACE-MATPES-r2SCAN-0 model as the calculator, three independent 1 ns MD trajectories were obtained at different temperatures (330 K, 400 K, and 500 K). Higher-temperature simulations were proven to enhance sampling of the configurational space of the target system.[S12] Second, 2,500 snapshots were uniformly extracted from each trajectory, yielding a dataset of 7,500 configurations. Third, three independent PiNet2 models were pretrained on this dataset with different random splittings (training:validation = 8:2). A total of 5 million gradient descent steps were performed using the Adam optimizer[S13] with a batch size of 1. The learning rate was reduced from 0.00005 every 100,000 steps at a decay rate of 0.994. The hyperparameters of PiNet2 models are listed in Table S1.

Table S1: Hyperparameters of Zero-field PiNet2 models.

Layer	Architecture	Parameter	Value
PI	[16] × 10	R_c	6.0 Å
II	[16,16,16,16]	GC blocks	5
PP	[16,16,16,16]	n_{basis}	10
Output	[16,1]	e_loss_multiplier	0.1
		f_loss_multiplier	100.0

This step leads to the PiNet2-prior models with the validation RMSEs for energy per atom and force are 0.5±0.1 meV/atom and 28±2 meV/Å. Then, we subsequently assessed the quality of PiNet2-prior model against the MACE-MATPES-r2SCAN-0 foundation model. To check this, three PiNet2-prior models were employed to run 1 ns NVT simulations at 330 K on the target system. As shown in Figure S3, the radial distribution functions (RDFs) of

different atomic species obtained from the PiNet2-prior models are in excellent agreement with those calculated using the corresponding foundation model. We also computed the MSDs of oxygen species (including those associated with hydronium ions) and proton. As we can see from the Figure S4 , the MSD curves predicted by the PiNet2-prior models closely reproduce those obtained from MACE-MATPES-r2SCAN-0. The estimated self-diffusion coefficients of oxygen atoms are $1.05 \pm 0.08 \times 10^{-9}$ m^2/s, which almost the same as those from one obtained from the MACE-MATPES-r2SCAN-0 (1.07×10^{-9} m^2/s).

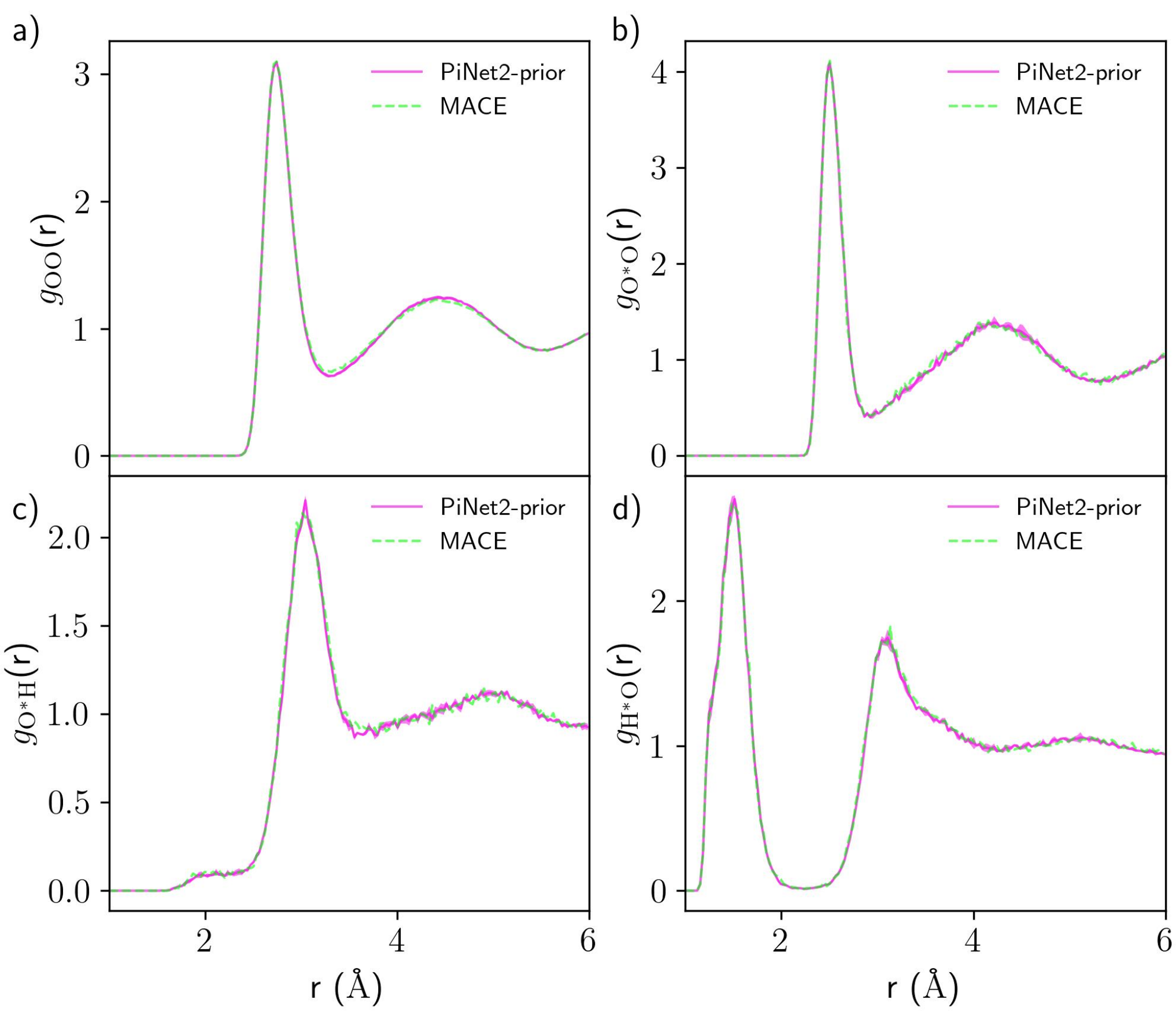

Figure S3: Radial distribution functions $g(r)$ obtained from 1 ns MD simulations at 330 K using PiNet2-prior models, alongside reference results from the MACE-MATPES-r2SCAN-0 foundation model. Superscripts * identify atoms in the hydronium ion, while other atoms are associated with water molecules. (a) Distribution of O atoms around O atoms; (b) Distribution of O atoms around O^* atoms; (c) Distribution of H atoms around O^* atoms; (d) Distribution of O atoms around H^* atoms. The standard deviations of PiNet2-prior models are shown in transparent colors.

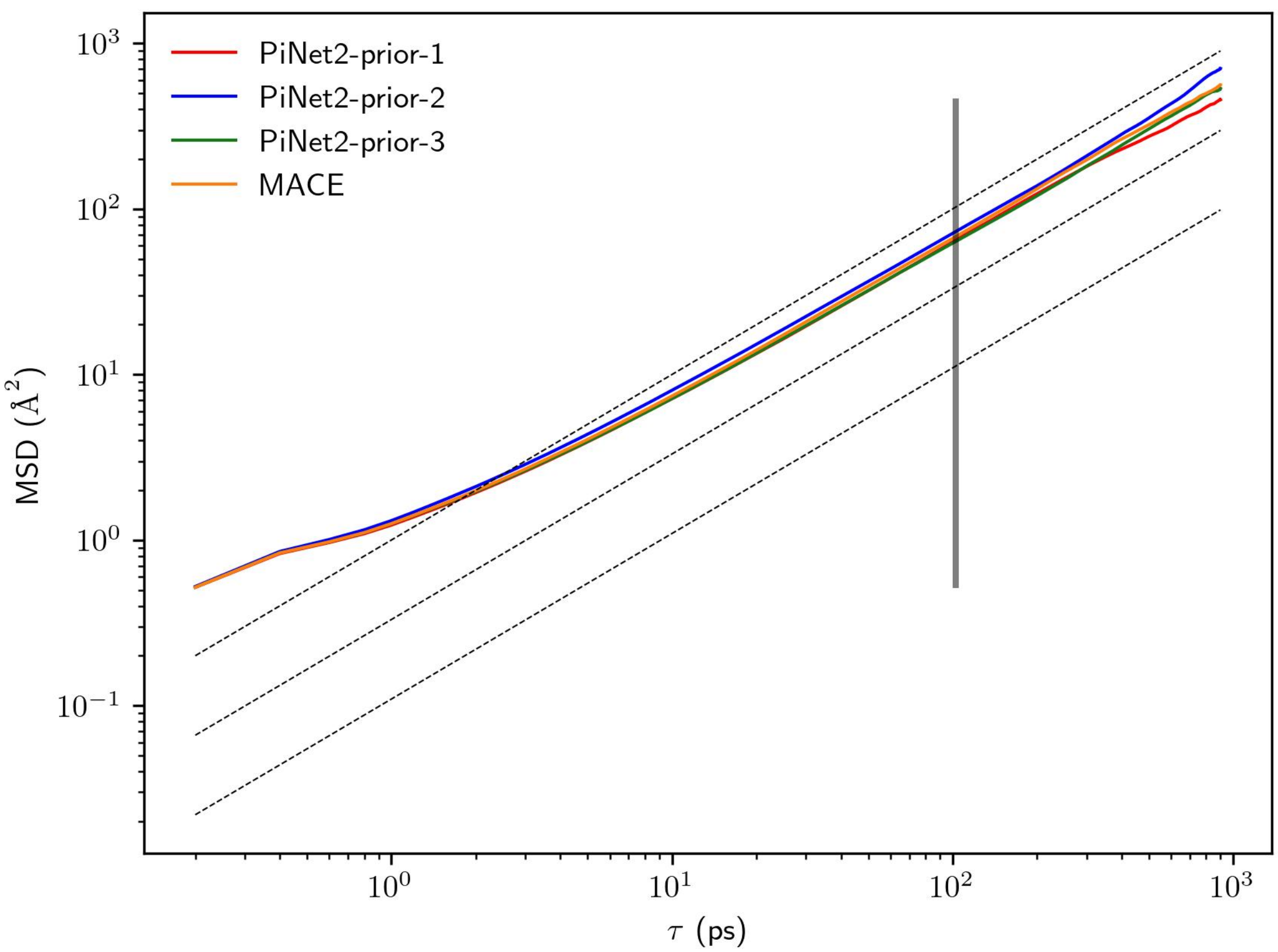

Figure S4: Mean squared displacements of all oxygen atoms, including those associated with hydronium ions, obtained from 1 ns MD simulations at 330 K using the PiNet2-prior models. The dashed lines correspond to $\log(\mathrm{MSD})/\log(\tau)=1$, and the shaded black region indicates the fitting window used to extract the diffusion coefficients.

4.2 Fine-tuning with PiNNAcLe-FT

PiNNAcLe-FT adopts an iterative active-learning strategy, where each iteration, termed a generation, comprises three key stages: configuration sampling, data labeling, and model training. During the sampling stage, MD simulations are carried out using the latest PiNet2 model over a predefined time scale, from which a number of unlabeled snapshots (denoted as sp_points) is uniformly extracted along the trajectory. In the subsequent labeling stage, these sampled configurations are evaluated at the target DFT level and employed for convergence assessment.

Model convergence in each generation is mainly determined on four criteria: the maximal absolute difference of energy per atom between predictions and labels (EMAX), the root mean squared errors (RMSE) of energy per atom (ERMSE), the maximal absolute difference of force components (FMAX), and the RMSE of atomic forces (FRMSE). The model training is only triggered for a number of training steps (retrain_step) and the atomic dress[S12] will be updated, if one of the newly labeled snapshot fails any of these four criteria. Otherwise, the newly labeled configurations are incorporated into the dataset accumulated from previous generations. Then, the current model will be used in the configuration sampling stage with a simulation time multiplied by a scaling factor (acc_fac) in next generation. Notably, the sampling time is capped by a chosen upper bound (max_time) to avoid excessively long trajectories. In this work, the sp_points, EMAX, ERMSE, FMAX, FRMSE, acc_fac, max_time, and retrain_step were set as 10, 2.5 meV/atom, 1 meV/atom, 500 meV/Å, 31 meV/Å, 2.0, 5000 ps and 5000 steps respectively.

A typical fine-tuning curves for a PiNNAcLe-FT run is shown in Figure S5. We have carried out three independent PiNNAcLe-FT runs, which lead to three PiNet2-ft models. The final validation RMSEs for energy and forces are 0.5±0.1 meV/atom and 25.8±0.9 meV/Å respectively. It is worth noting that all PiNNAcLe-FT runs were completed within fewer than 25 generations, corresponding to less than 250 single-point DFT calculations and 80,000 training steps.

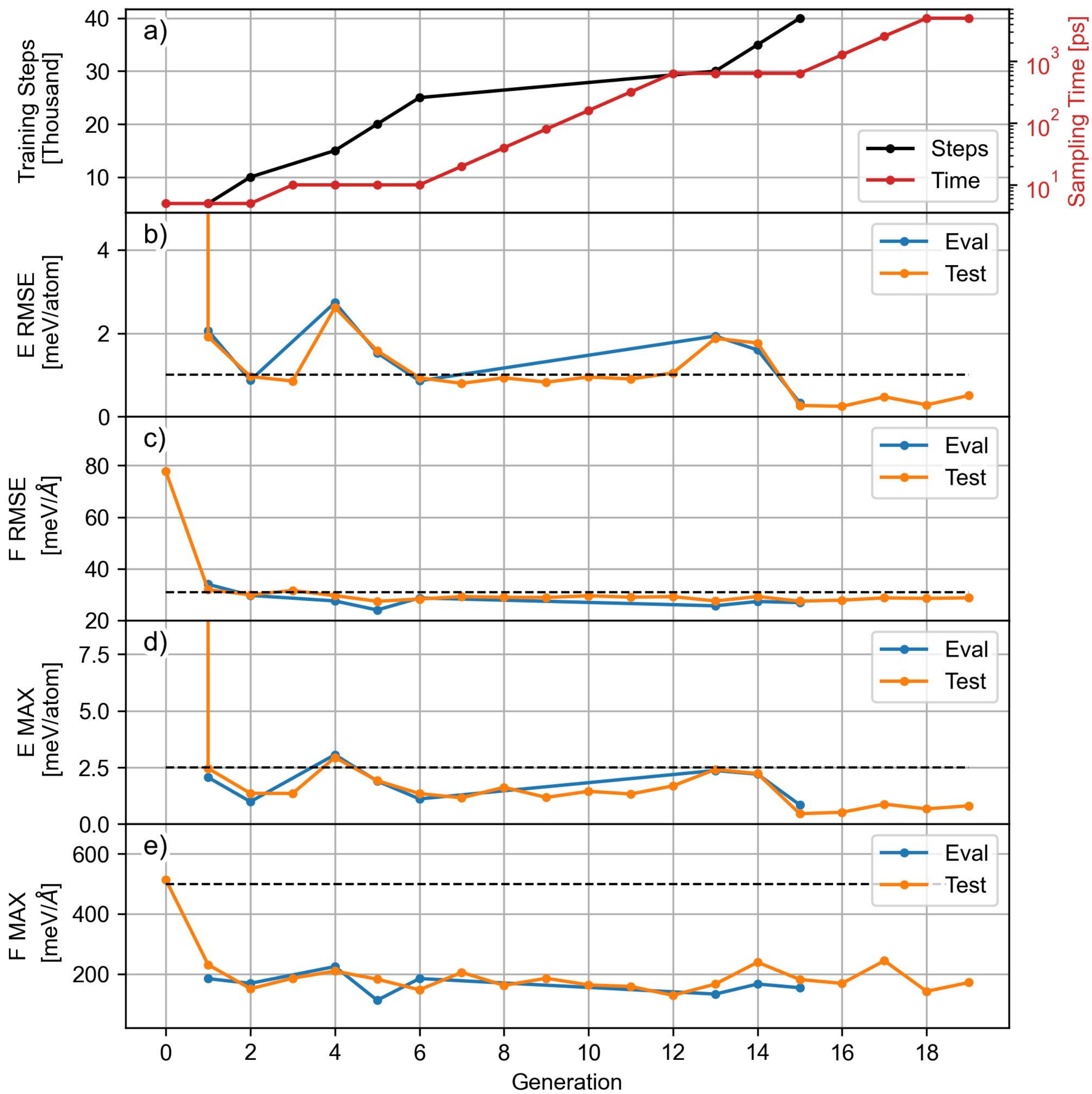

Figure S5: Fine-tuning curve of the second PiNet2-prior model in the PiNNAcLe-FT task at the r^2SCAN/TZV2P DFT level. (a) Accumulated training steps and time scale of the sampled trajectory; (b,c) energy and force errors on the validation data during the training stage and the test data during the sampling stage; (d,e) EMAX and FMAX on the validation data during the training stage and the test data during the sampling stage. Dashed lines are the corresponding tolerances.

5 Water and proton self-diffusion coefficients from PiNet2-ft models

To analyze the dynamical properties of the excess proton, the position of the hydronium ion was used as an approximate coordinate of the proton, which is a common and well-established practice in molecular simulations.[S10,S14] The proton displacement between two neighbor frames was computed as

$$\Delta\mathbf{r}(t) = \mathbf{r}(t+1) - \mathbf{r}(t) - \mathbf{L} \cdot \mathrm{round}\left(\frac{\mathbf{r}(t+1) - \mathbf{r}(t)}{\mathbf{L}}\right), \tag{1}$$

where $\mathbf{r}(t)$ denotes the position of the oxygen atom in the hydronium ion at time t, and $\mathbf{L}$ represents the simulation box. The minimum-image convention was applied to account for periodic boundary conditions. The mean squared displacement (MSD) of species i was calculated as $\langle|\mathbf{r}_i(t) - \mathbf{r}_i(0)|^2\rangle$ by using the tidynamics package.[S15] From the linear regime of the MSD, the self-diffusion coefficient (D) is extracted via the Einstein relation:

$$D = \lim_{t\to\infty} \frac{\mathrm{MSD}(t)}{2dt}. \tag{2}$$

, where d is the number of spatial dimensions considered in the diffusion analysis.

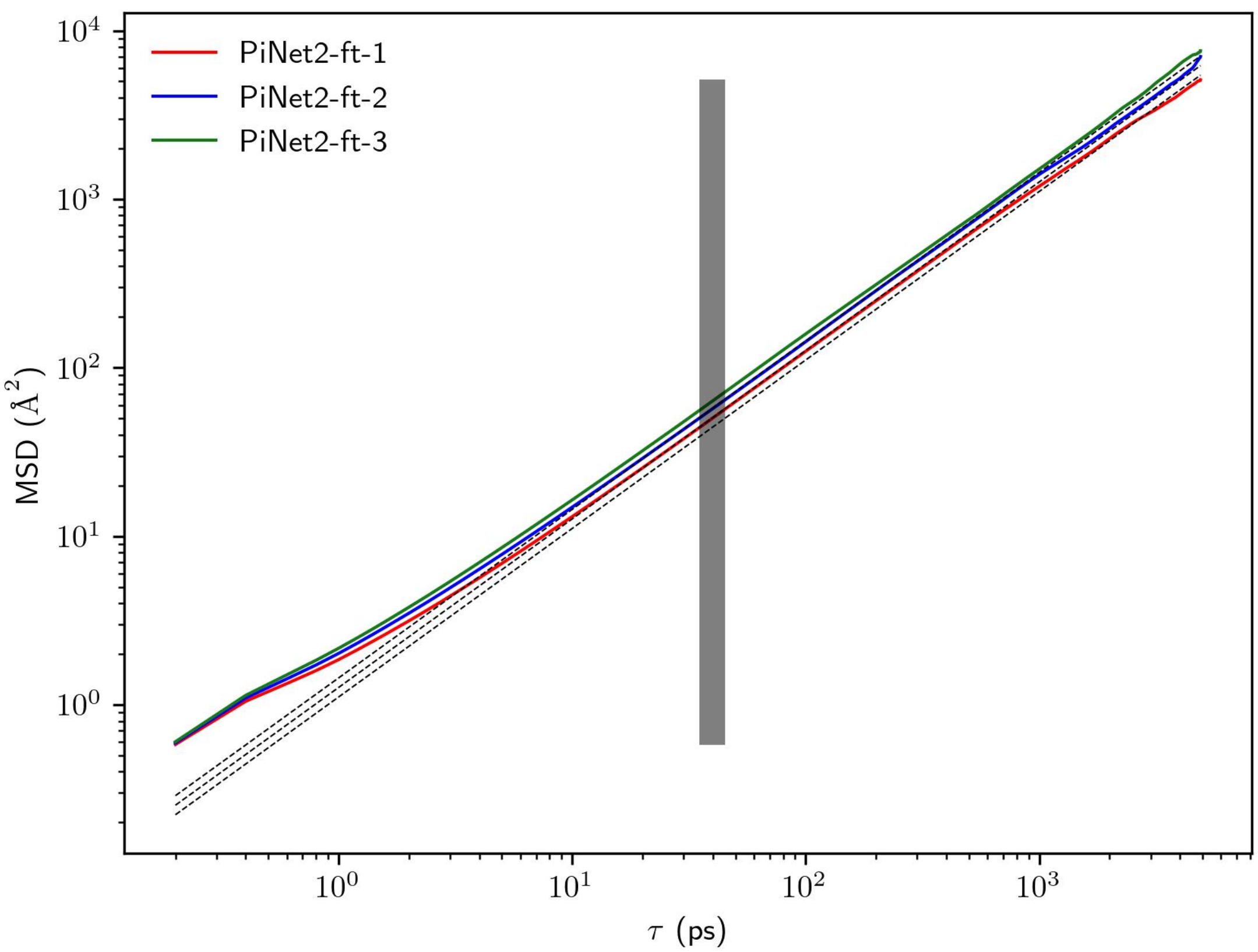

Figure S6: Mean squared displacements of all oxygen atoms, including those associated with hydronium ions, obtained from 5 ns MD simulations at 330 K using the PiNet2-ft models. The dashed lines correspond to $\log(\mathrm{MSD})/\log(\tau)=1$, and the shaded black region indicates the fitting window used to extract the diffusion coefficients.

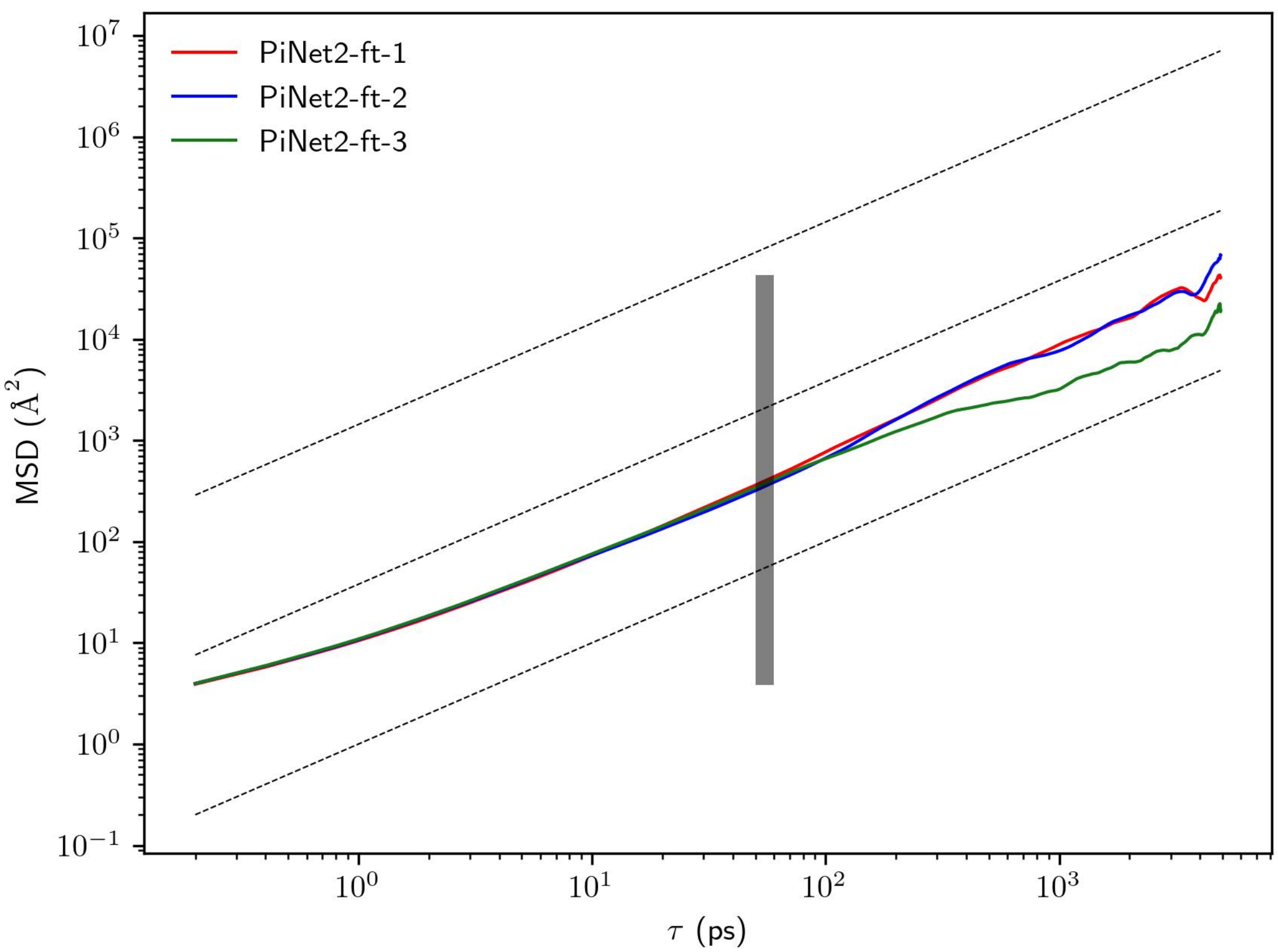

Figure S7: Mean squared displacements of the proton obtained from 5 ns MD simulations at 330 K using the PiNet2-ft models. The dashed lines correspond to log(MSD)/ log(τ)=1, and the shaded black region indicates the fitting window used to extract the diffusion coefficients.

6 Finite-field MLMD with AD(OS) models

6.1 Finite field perturbation formula

The first order expansion of the AIMD finite field Hamiltonian[S16,S17] under electric field E_β in direction β is

$$H(E) \simeq H_{\mathrm{E}=0} - \sum_{\beta} M_{\beta,\mathrm{E}=0} E_\beta \tag{3}$$

where $H_{\mathrm{E}=0}$ and $M_{\mathrm{E}=0,\beta}$ are the Hamiltonian and dipole moments calculated at zero field. The resulting force components $F_{i,\alpha}$ on atom i are

$$F_{i,\alpha} \simeq F_{i,\alpha}^{\mathrm{E}=0} + \sum_{\beta} \frac{\partial M_{\beta,\mathrm{E}=0}}{\partial r_{i,\alpha}} E_\beta \tag{4}$$

where $r_{i,\alpha}$ is the nuclear position coordinate. Hence the force at each field strength depends on the Born effective charge (BEC) tensor which by definition[S18] is

$$Z_{\alpha,\beta}^{E=0} = \frac{\partial M_{\beta,\mathrm{E}=0}}{\partial r_{i,\alpha}} = \left.\frac{\partial F_{i,\alpha}}{\partial E_\beta}\right|_{\mathrm{E}=0}. \tag{5}$$

6.2 AD(OS) model for dipole and BEC predictions

The PiNet2-dipole/AD(OS) model was previously introduced to model the dipole moment of condensed phase systems.[S19] The PiNet2 equivariant architecture contains 3-dimensional atomic feature vectors and their outputs ${}^{3}\mathbb{P}_i$ are here interpreted as atomic dipole (AD) vectors. In addition the dipoles from the formal oxidation states (OS) of the atoms are added to output the total dipole of the system:

$$M^{\mathrm{pred}} = \sum_{i} {}^{3}\mathbb{P}_i + q_i^{\mathrm{OS}} r_i. \tag{6}$$

For the protonated water system, the OS is assigned to be +1 for a hydrogen atom and -2 for oxygen atom.

This dipole model can be used to predict also the BEC tensor by calculating the gradient of the predicted dipole moment:

$$Z^{\text{pred}}_{i,\alpha\beta} = \frac{\partial M^{\text{pred}}_{\beta}}{\partial r_{i,\alpha}}. \tag{7}$$

Compared to the perturbed neural network potential (PNNP),[S20] both the dipole and the BEC are described consistently with the AD(OS) model in this work.

6.3 Dataset generation for the protonated water system

A set of bulk geometric conformations were selected from a trajectory containing 64 water molecules and an added solvated proton. These snapshots were taken from a trajectory propagated by a PiNet-2 model fine-tuned as described above. Snapshots were collected at intervals of 200 ps across a 5 ns trajectory. For these geometries, single point calculations using the r^2SCAN functional were performed both at zero macroscopic field and with electric fields of 0.0001 a.u. applied in one spatial dimension at a time. The same density functional theory parameters were used as in the fine-tuning described above. Maximally localized Wannier functions were used to calculate the supercell dipole moment for each frame of the data set, and forces obtained at zero and finite electric fields were used to calculate the differentials of the Born effective charge matrix.

6.4 Model training on the protonated water dataset

For the learning of Born effective charges and supercell dipole moments, the constructed solvated proton data set was split into training and test sets in a 4:1 ratio. AD(OS) models were trained on the training part of the data set until convergence, which accounted to 1×10^6 training steps. The hyperparameters used in the AD(OS) model training are listed in Table S2. To show the necessity of the oxidation state feature of the model, Figure S9 shows the attempted training of an AD-model that does not include this oxidation state information. The AD model cannot capture the system dipoles' dependence on the position

of the excess proton. This flaw leads to unsuccessful training and eventually overfitting of both the system dipole and the Born effective charges. Prediction of the latter is likely failing due to the differential relationship between the BEC and the system dipole (Equation 7).

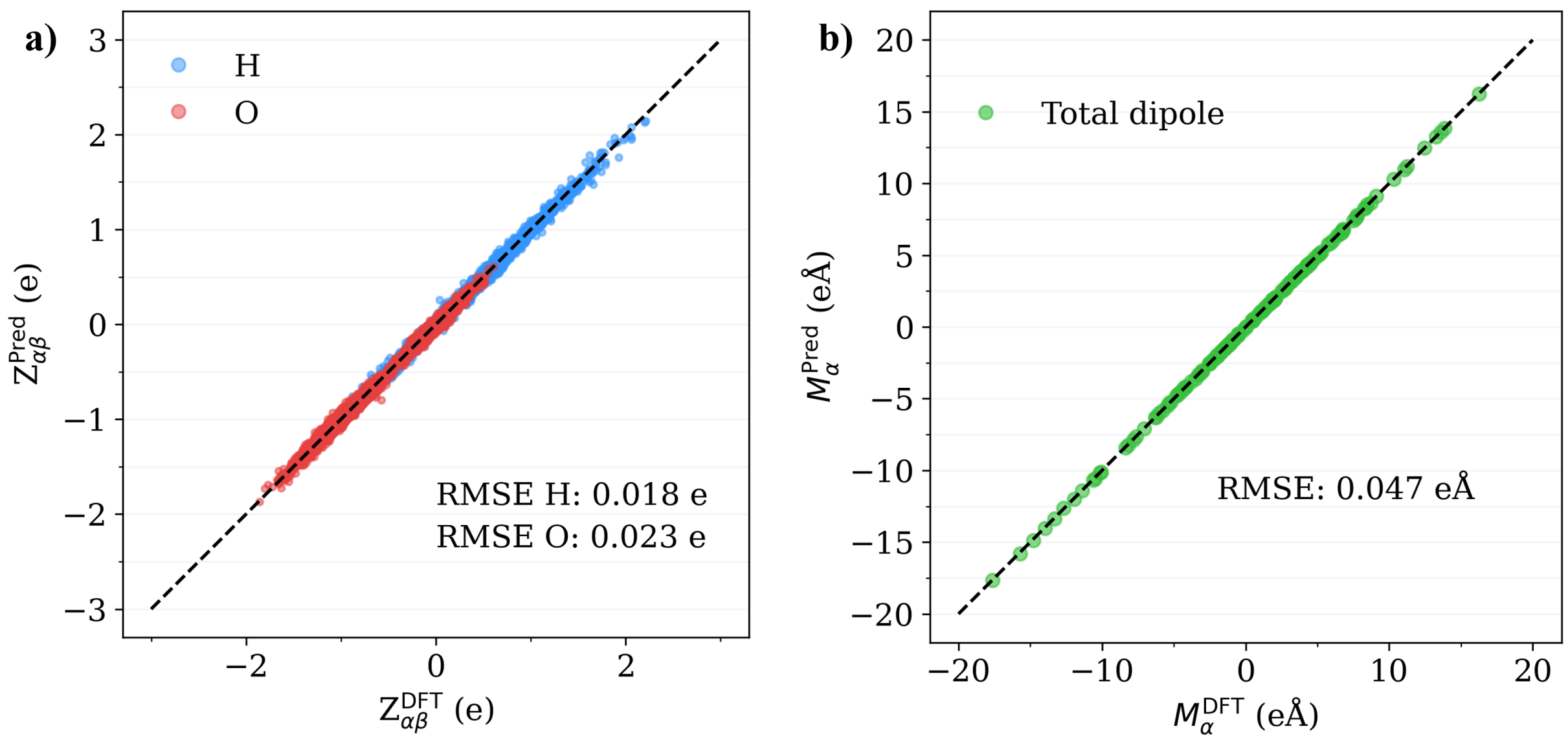

Figure S8: Parity plots showing the accuracy of BEC and the total dipole prediction for the protonated water dataset.

Table S2: Hyperparameters used to train the PiNet-dipole AD(OS) models on the liquid water dataset. R_c represents the interaction cut-off, and n_{basis} is the number of basis functions for each (i,j) tuple.

Layer	Architecture	Parameter	Value
PI	[16]×10	R_c	4.5 Å
II	[16, 16]	GC blocks	4
PP	[16, 16]	n_{basis}	10
Output	[16, 1]		

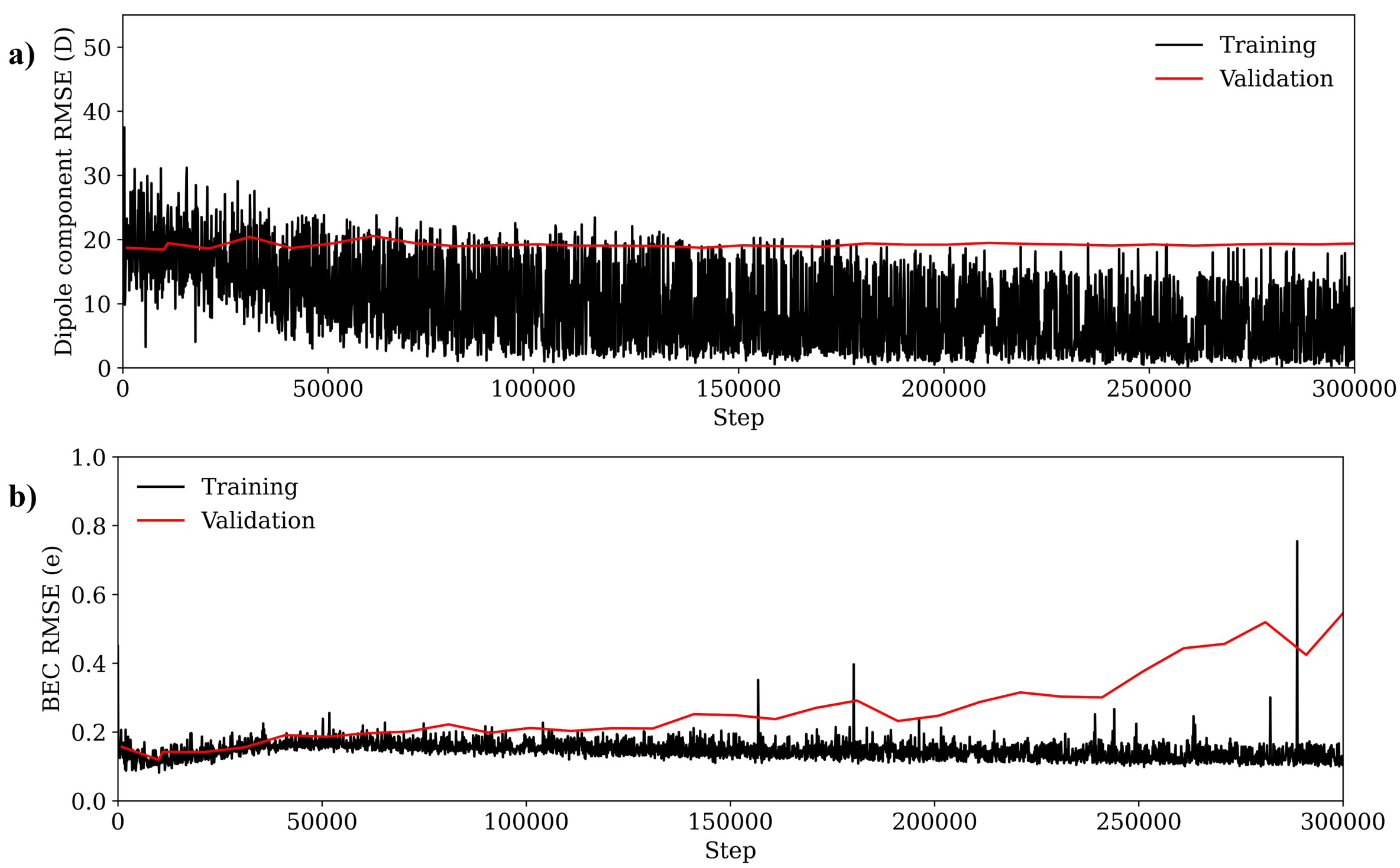

Figure S9: Training log for attempted training of an AD-model on the protonated water dataset.

7 Convergence plots of proton drift velocity in finite-field MLMD

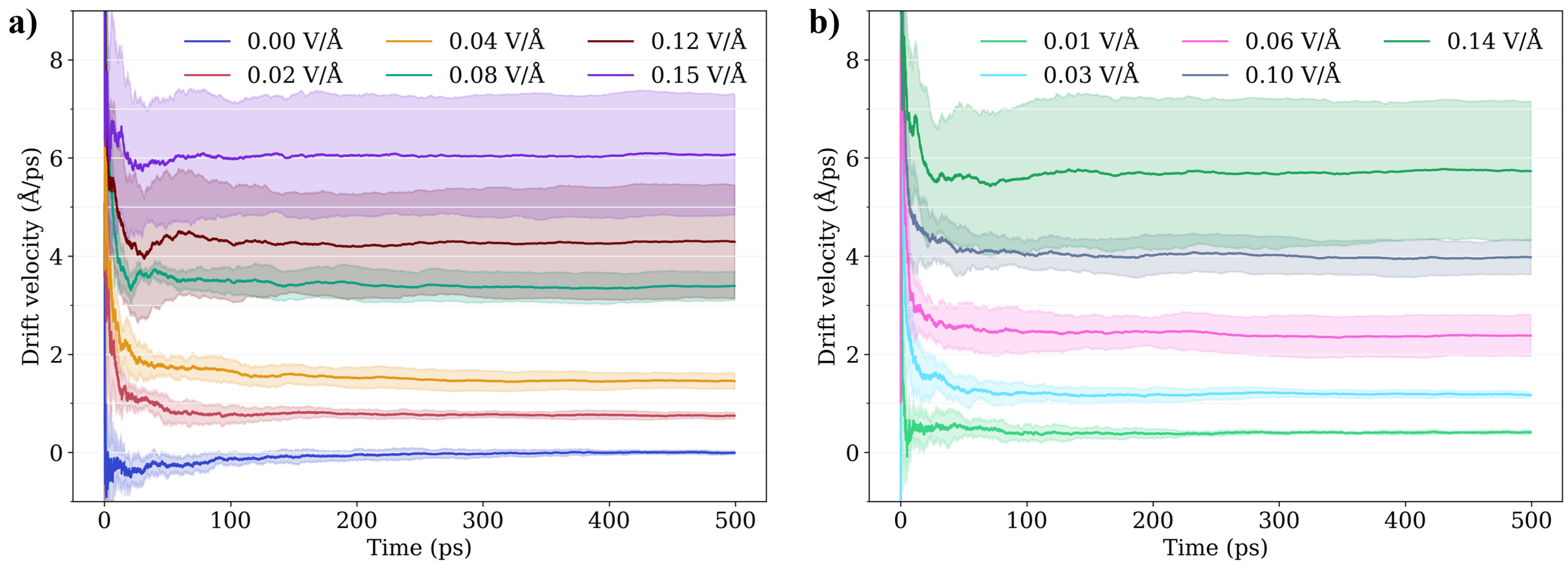

Figure S10: Cumulative average of the drift velocity of the aqueous proton under influence of macroscopic electrostatic fields. Each line is the average of six independent trajectories, and the shaded regions mark one standard deviation. To increase visibility of otherwise highly overlapping confidence regions, the data have been split into two subfigures.

8 Finite size effect on the dynamical properties

Evidently, the addition of a protonic defect changes the water structure, although fully capturing this effect is made difficult by a high degree of structural variation. One can also consider the relatively small simulation system (64 water molecules) used in this work. A small system is subject to finite-size effects, which can both be physical effects that would arise in a confined system, and simulation artifacts due to the periodic boundaries of a simulation. Additionally, in our study, the size of the system determines the proton concentration, which has its own effect on the simulation outcome. To get an idea of the sizes and characteristics of these various effects, the non-equilibrium molecular dynamics simulations were repeated with a system of 128 water molecules and one solvated proton. Once again, six independent trajectories were generated. For these new simulations, drift velocities are shown in Figure $S11$. While the drift velocities lie relatively close to those of the smaller system, the apparent linear behavior is weaker and the error bars remain large.

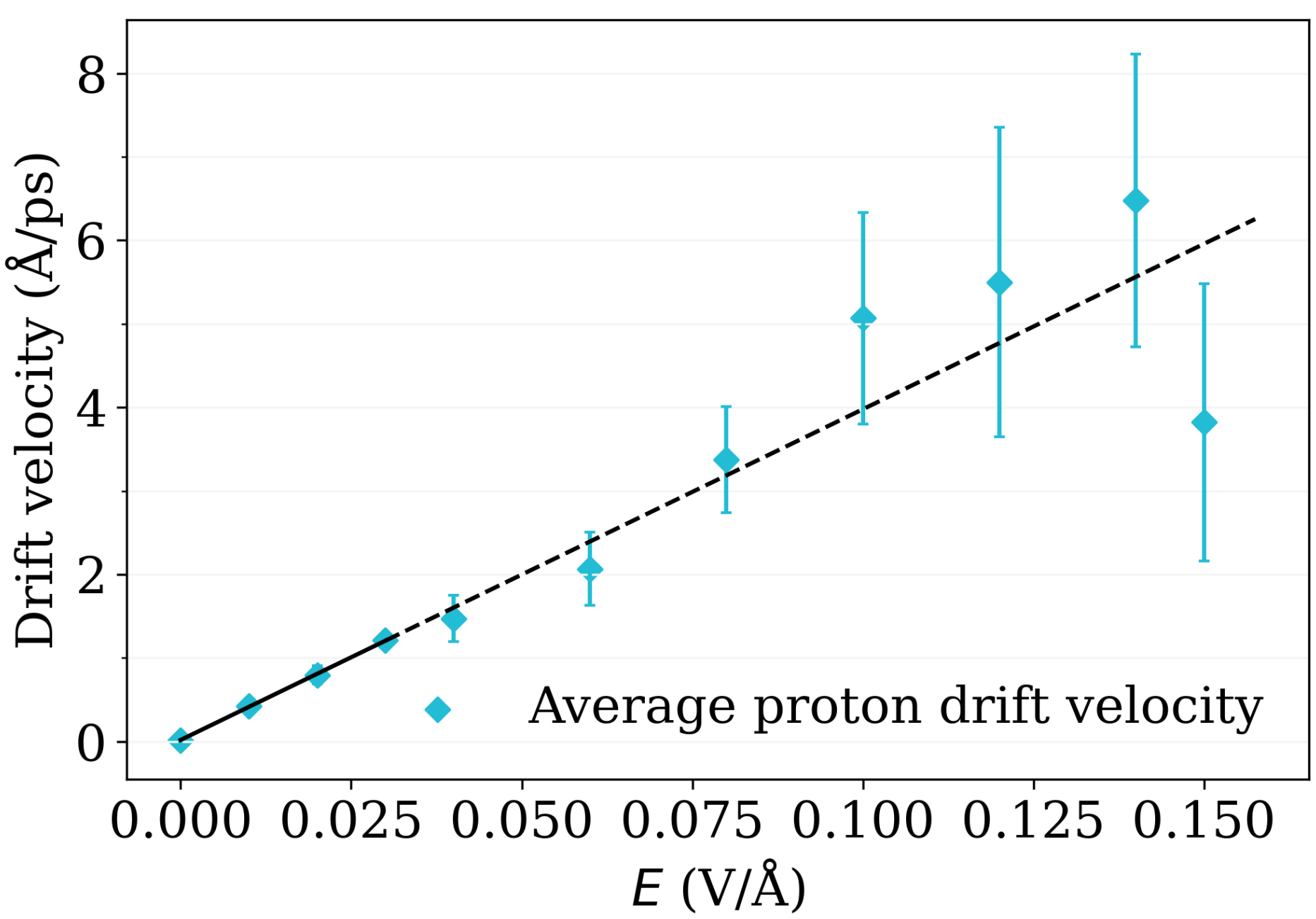

Figure S11: Drift velocity of the aqueous proton as a function of electric field E for a system of 128 water molecules and one solvated proton. Error bars denote one standard deviation.

The linear fit to the low-field (E = 0.00 - 0.03 V/Å) drift velocity corresponds to a diffusion coefficient of 11.3 ± 0.4 $\times 10^{-9}$ m^2/s.

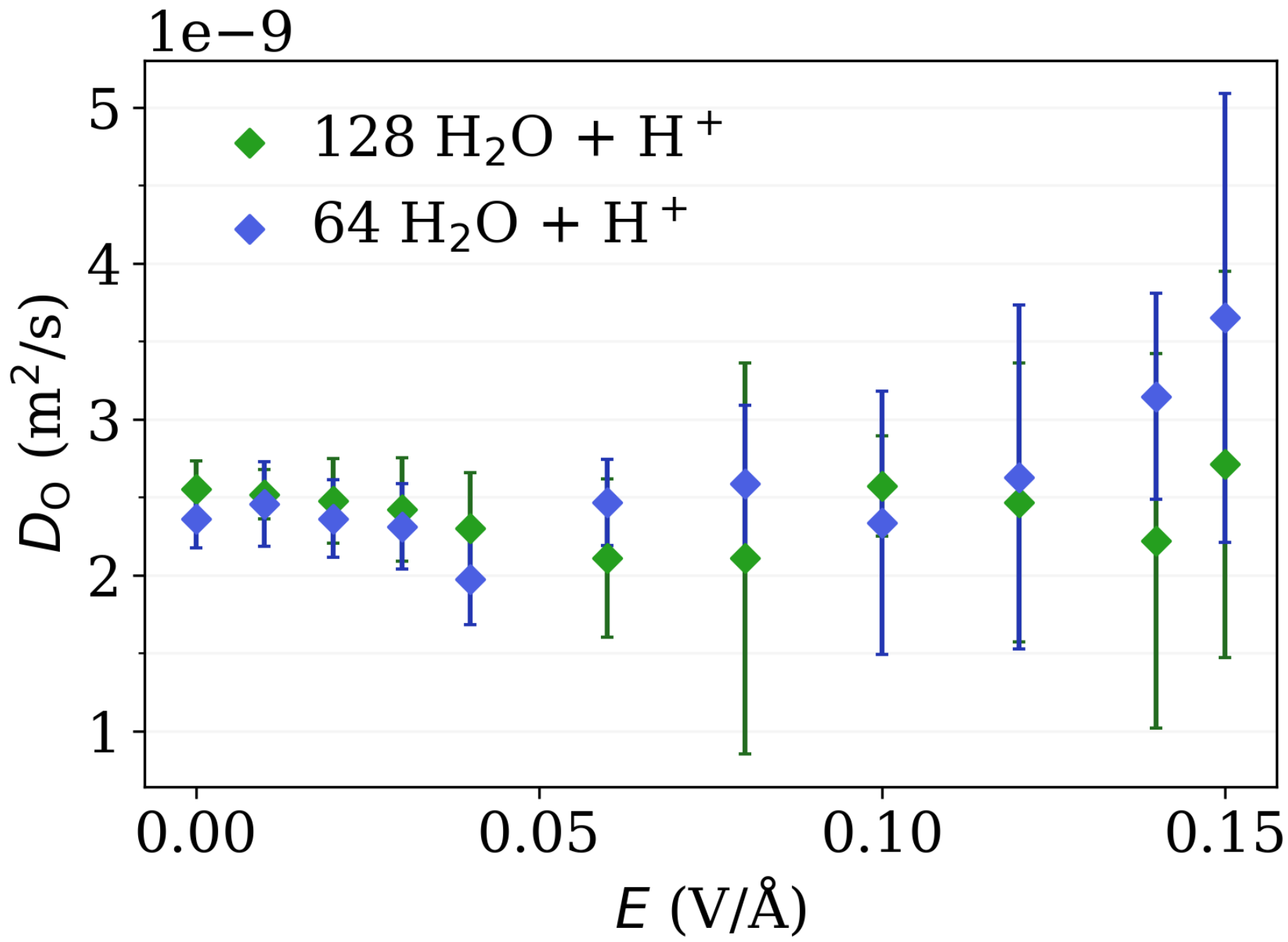

Figure S12: The field-dependent self-diffusion coefficient of oxygen atoms in protonated water with two different system sizes.

Looking at the changes in the water self-diffusion coefficient for protonated water when the system size is increased (Figure S12), we see that the averages remain close to the zero field diffusivity throughout the whole field range. However, the uncertainty continues to be very large.

9 H-bond analyses and tetrahedral order parameters

To consolidate and further explore the structural diversity of simulated neat and protonated water structures, the coordination environment of water molecules was investigated through 1) the number of hydrogen bonds that an average water molecule partakes in, and 2) the tetrahedral order parameter of the system.

The tetrahedral order coefficient was calculated from the angles θ_{jk} between all pairs within the four closest neighbors of a given water molecule according to Equation 8.[S21]

$$Q = 1 - \frac{3}{8} \sum_{j=1}^{3} \sum_{k=j+1}^{4} \left(\cos\theta_{jk} + \frac{1}{3} \right)^2 \tag{8}$$

The average distributions obtained across a range of field strengths are shown in Figure S13 below. Similarly to what has been reported in previous studies,[S22] the tetrahedral order is increasing with field strength.

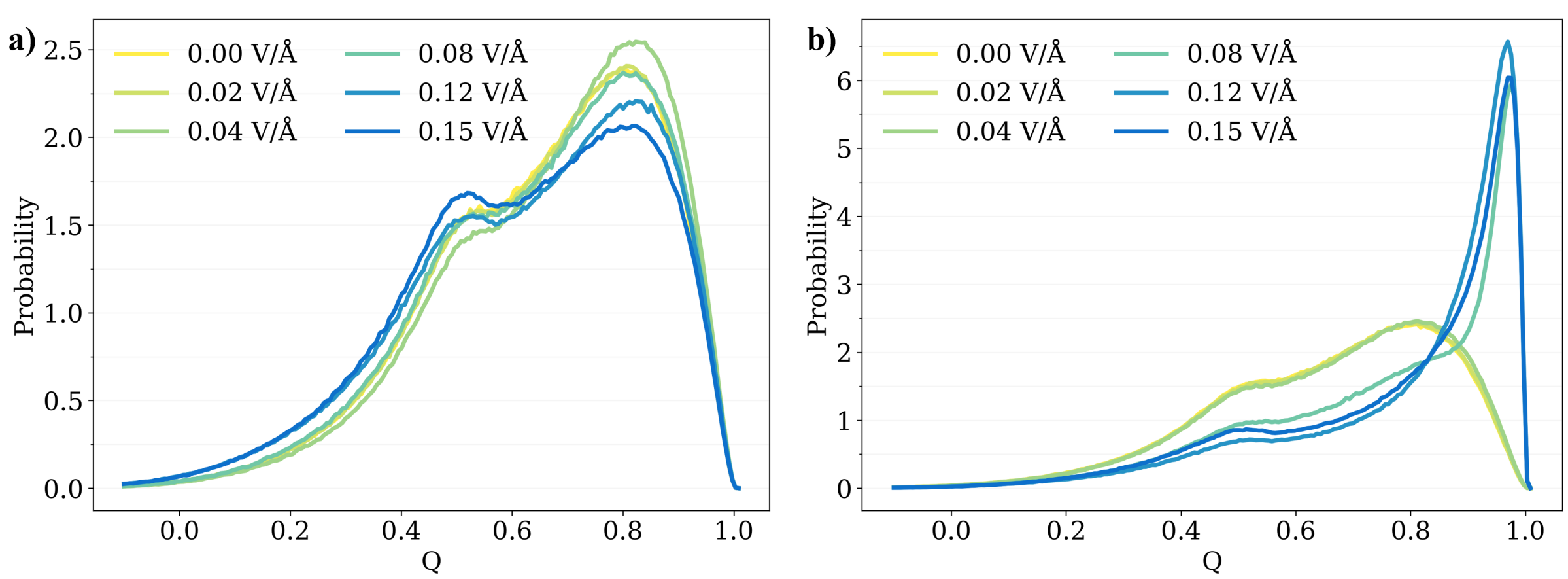

Figure S13: Distribution of the tetrahedral order parameter Q for a) protonated and b) neat water. Each line represent an average of six independent trajectories. The number of different field strengths represented has been reduced to increase readability.

Our investigation of the average number of hydrogen bonds per water molecule is summarized in Figure S14. Within this analysis, an interaction is classified as a hydrogen bond if the O-O distance is less than 3.5 Å and the O-O-H angle is less than 30 degrees. Closely

mirroring the oxygen self diffusion constant of the solutions, the average number of hydrogen bonds per water molecule increases with field strength, and the protonated water sees the same number of or even fewer hydrogen bonds on average, while all high-field data points show great variety between individual simulations.

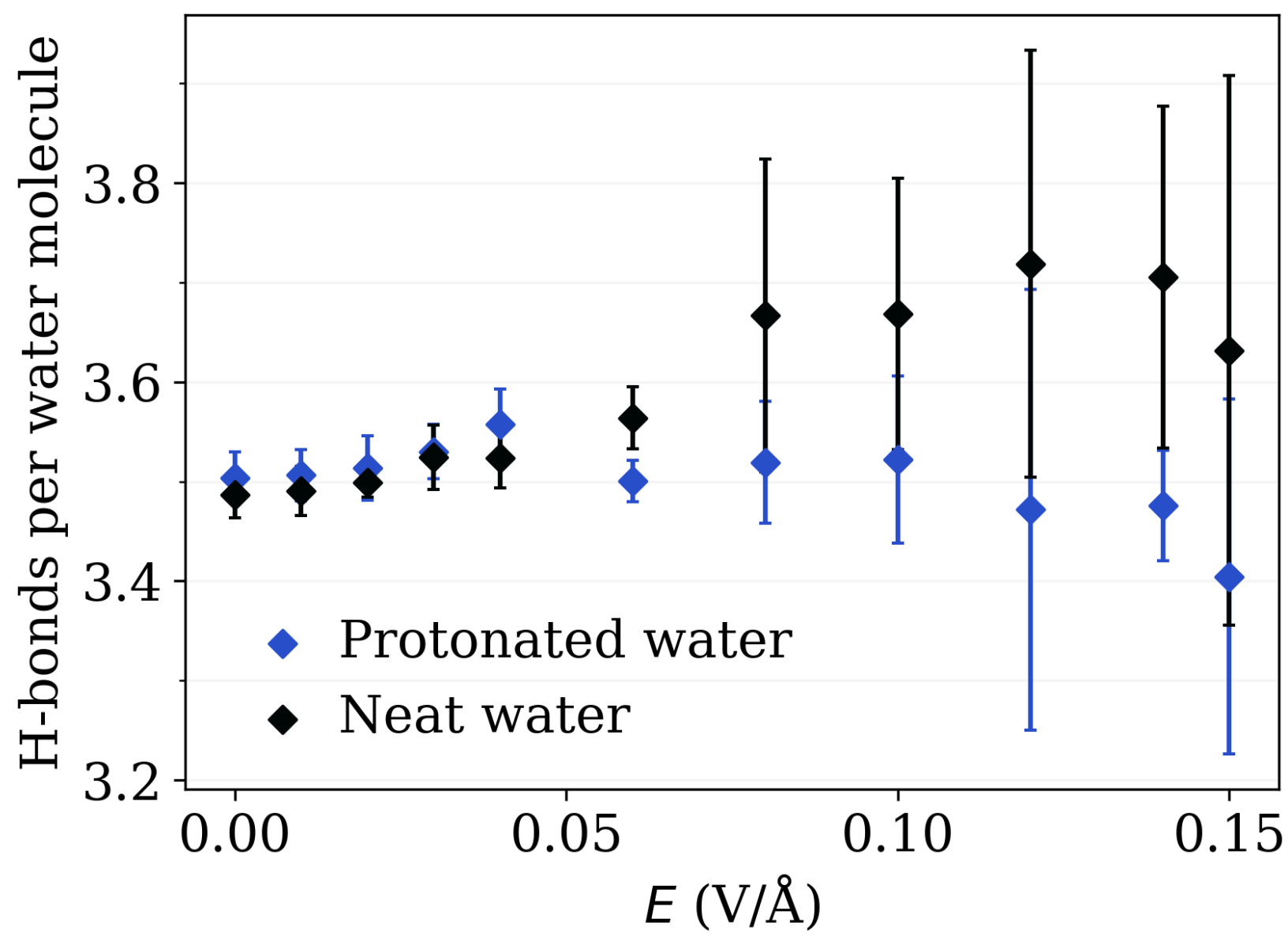

Figure S14: The average number of hydrogen bonds a water molecule partakes in, as function of electric field E. Neat water data is shown in black and protonated water data in blue.

10 Anisotropic analyses of water diffusion

The anisotropic self-diffusion coefficients of oxygen atoms in neat and protonated water under an applied electric field are shown in Figure S15. For oxygen diffusion in neat water, both $D_\mathrm{O}^\perp$ and $D_\mathrm{O}^\parallel$ decrease significantly when the electric field higher than 0.05 V/Å, indicating the onset of electrofreezing of the water molecules. Similar electrofreezing behavior has also been observed in AIMD simulations,[S23] further supporting the accuracy and reliability of the reactive ML potentials developed in the present work. Notably, $D_\mathrm{O}^\perp$ and $D_\mathrm{O}^\parallel$ remain nearly identical even at an electric field of 0.15 V/Å, suggesting that oxygen diffusion in neat water remains essentially isotropic despite the strong applied field. In contrast, oxygen diffusion in protonated water exhibits markedly different behavior. Both $D_\mathrm{O}^\perp$ and $D_\mathrm{O}^\parallel$ increase progressively with increasing electric field, reaching approximately 3.5×10^{-9} $\mathrm{m^2/s}$ at 0.15 V/Å. Moreover, a noticeable difference between $D_\mathrm{O}^\perp$ and $D_\mathrm{O}^\parallel$ emerges at high field strengths, indicating anisotropic oxygen diffusion induced by the electric field.

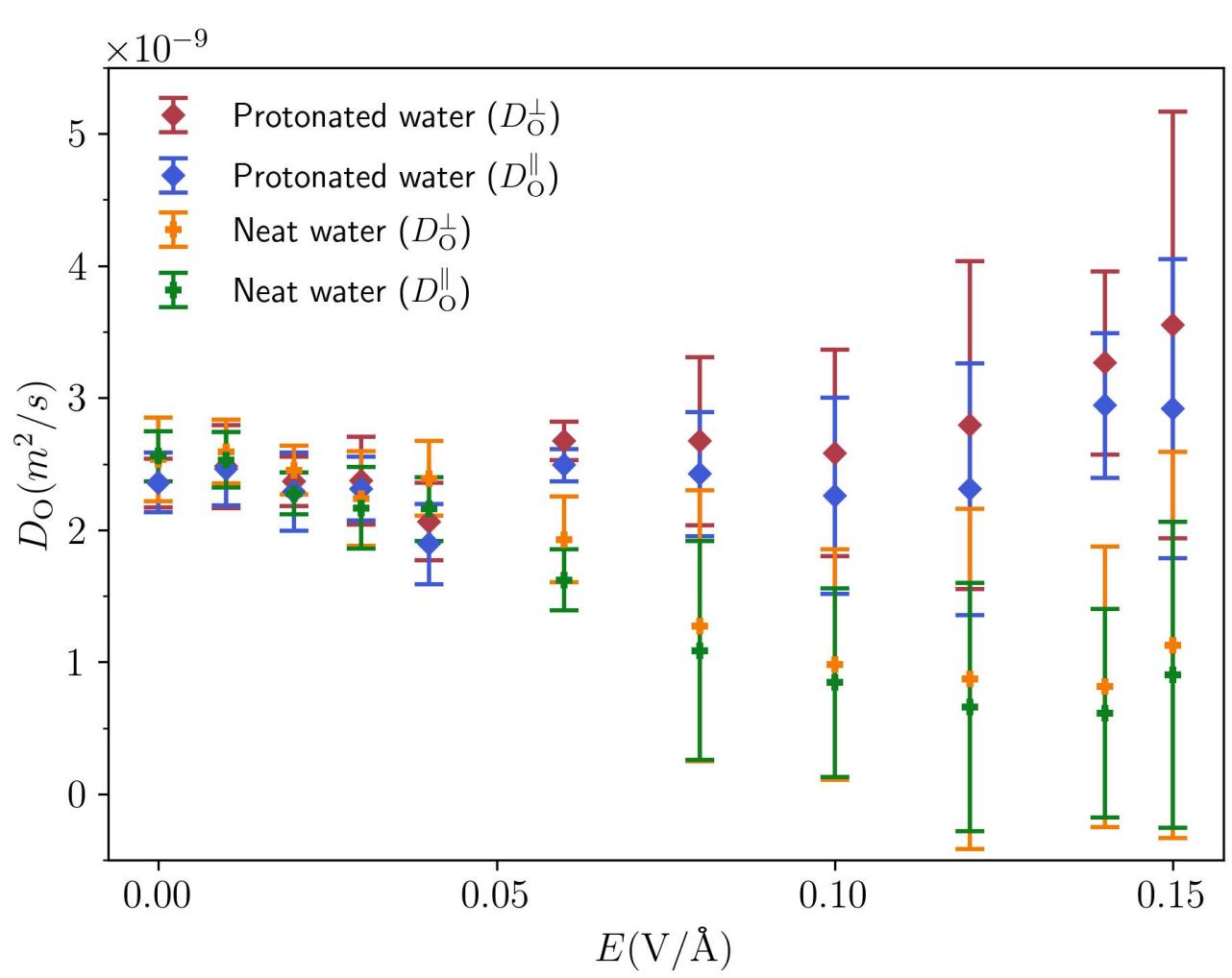

Figure S15: The anisotropic self-diffusion coefficients of oxygen atoms in neat water (64 H_2O) and protonated water (64 H_2O + 1 excess proton) systems Å under electric field. The $\perp$ and $\parallel$ denote the components perpendicular and parallel to the direction of the applied electric field. The error bars represents the cooresponding standard deviations.)

11 Structural heterogeneity at high field

The trajectories obtained from simulations under high electric fields show large structural diversity. As can be seen in the main manuscript, this diversity presents as a high variance in computed structural and dynamical parameters for both neat and protonated water. For example, while the neat water at 0.8-0.15 V/Å on average display lower oxygen self-diffusion than neat water (Figure 5 of the main manuscript), the values are accompanied by large error bars. This behavior is mirrored by an on-average decrease in the number of hydrogen bonds per water molecule (Figure S14) and a more structured O-O radial distribution function, both with large variation between independent trajectories under the same condition. As a complement to the field-averaged RDFs (Figure 4c-d) in the Main Text, we here show the radial distribution functions of all trajectories for protonated water at 0.12 V/Å and neat water at 0.15 V/Å (Figure S16) to exemplify the large variance. The nomenclature comes as follows: as mentioned before, we have fine-tuned three models (1, 2 and 3) with PiNNAcLe-FT and launched two independent simulations (a and b) for each model.

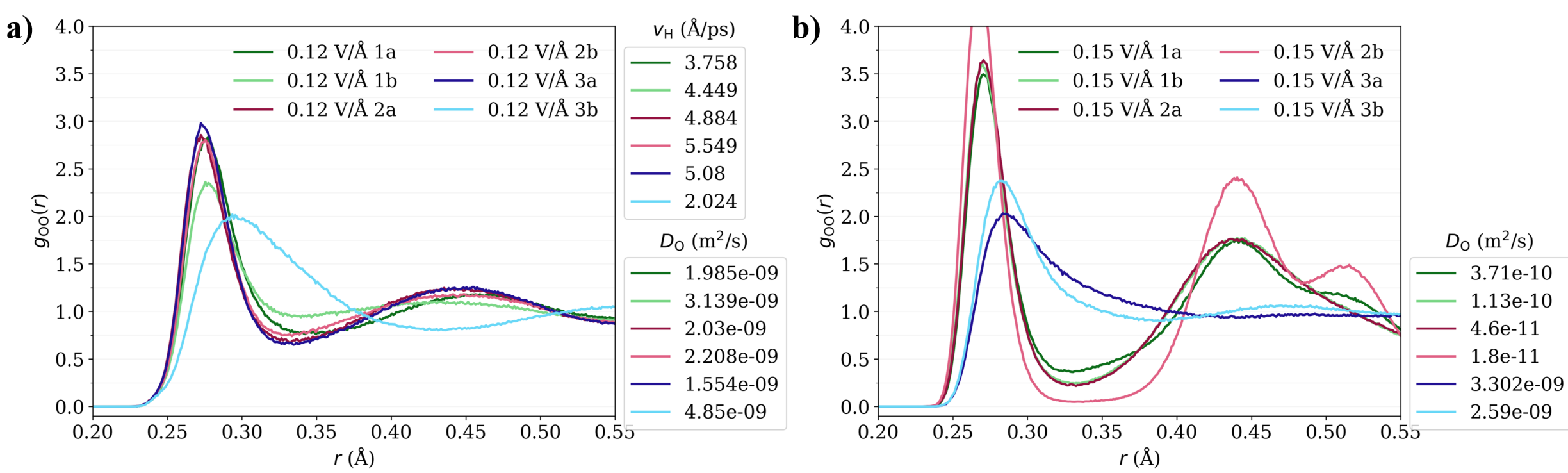

Figure S16: The O-O radial distribution functions for a) all trajectories of protonated water under $E = 0.12$ V/Å, and b) all trajectories of neat water under $E = 0.15$ V/Å. The number 1, 2, and 3 in the trajectory label denote the PiNet-ft model that was used for prediction of zero-field forces. To the right of the radial distribution functions, values of the proton drift velocity ν_{H} and the oxygen self diffusion coefficient D_{O} for the individual trajectories are presented.

12 Accuracy assessments of MLIP at high field

The accuracy of predicted forces, both zero field and field-induced, need to be preserved also at the high fields applied throughout the study. In addition, since a high structural variety has been observed at high field strengths, it is crucial to confirm that these states are equally well described by our machine learning models. To validate the predictions, three trajectories of protonated water simulated at 0.12 V/Å (Figure S16a) and three neat water trajectories at 0.15 V/Å (Figure S16b) were chosen for detailed studies. It is worth noting that higher structural diversity was observed at 0.12 V/Å compared to 0.14 or 0.15 V/Å for the protonated water systems. From these trajectories, corresponding to trajectories "1a", "1b", and "3b" of Figure S16a and "1a", "2b", and "3a" of Figure S16b, 20 evenly spaced snapshots were selected in the time-range 100-500 ps. Then, atomic forces, Born effective charges, and total dipoles for these snapshots were evaluated using CP2K with the r^2SCAN functional.

As shown in Figures S17 and S18, both our models for total forces and Born effective charges maintain prediction accuracy at high field strengths. Evidently, fluctuations in the zero-field training data contain enough information for the models to infer the properties of globally polarized configurations.

From Figure S19 it can be seen that dipole predictions become slightly less accurate for the highly polarized, ice-like, geometries of trajectory "2b" of water at 0.15 V/Å, that are also partially sampled in trajectory "1a" of the same system. This error is still negligible (about 3%) compared to the magnitude of total dipole moments ($\approx$40 eÅ) and their natural fluctuations. Additionally, for a finite E-simulation, the accuracy of the predicted dipoles is not directly affecting the system dynamics.

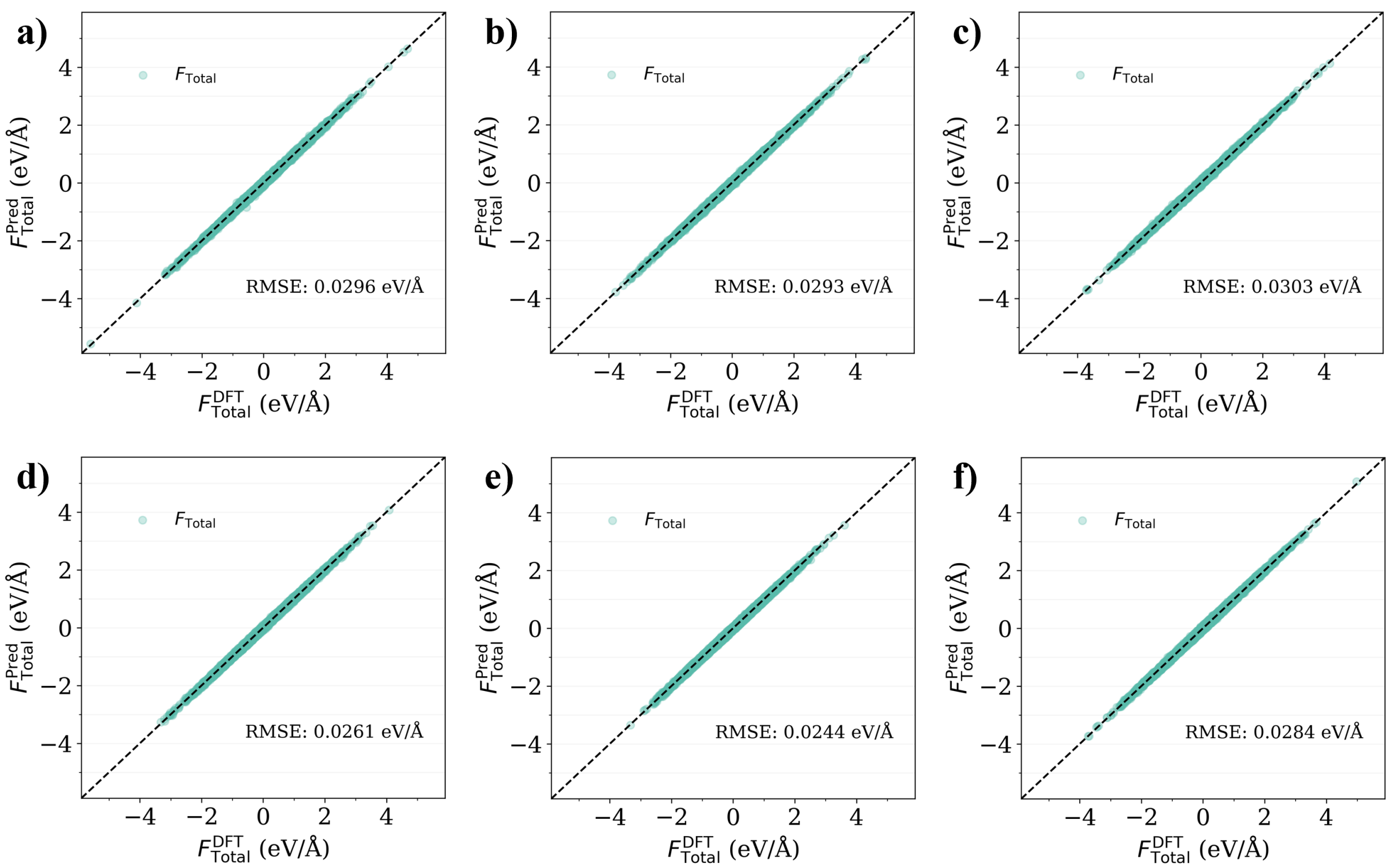

Figure S17: The prediction accuracy of total forces evaluated by summing the predicted zero-field and field perturbation components against the corresponding finite-field r^{2}SCAN calculations. Panels a), b), and c) show data from the three trajectories "1a", "1b", and "3b" of protonated water at $E = 0.12$ V/Å, while panels d), e), and f) show data from the trajectories "1a", "2b", and "3a" of neat water at E = 0.15 V/Å.

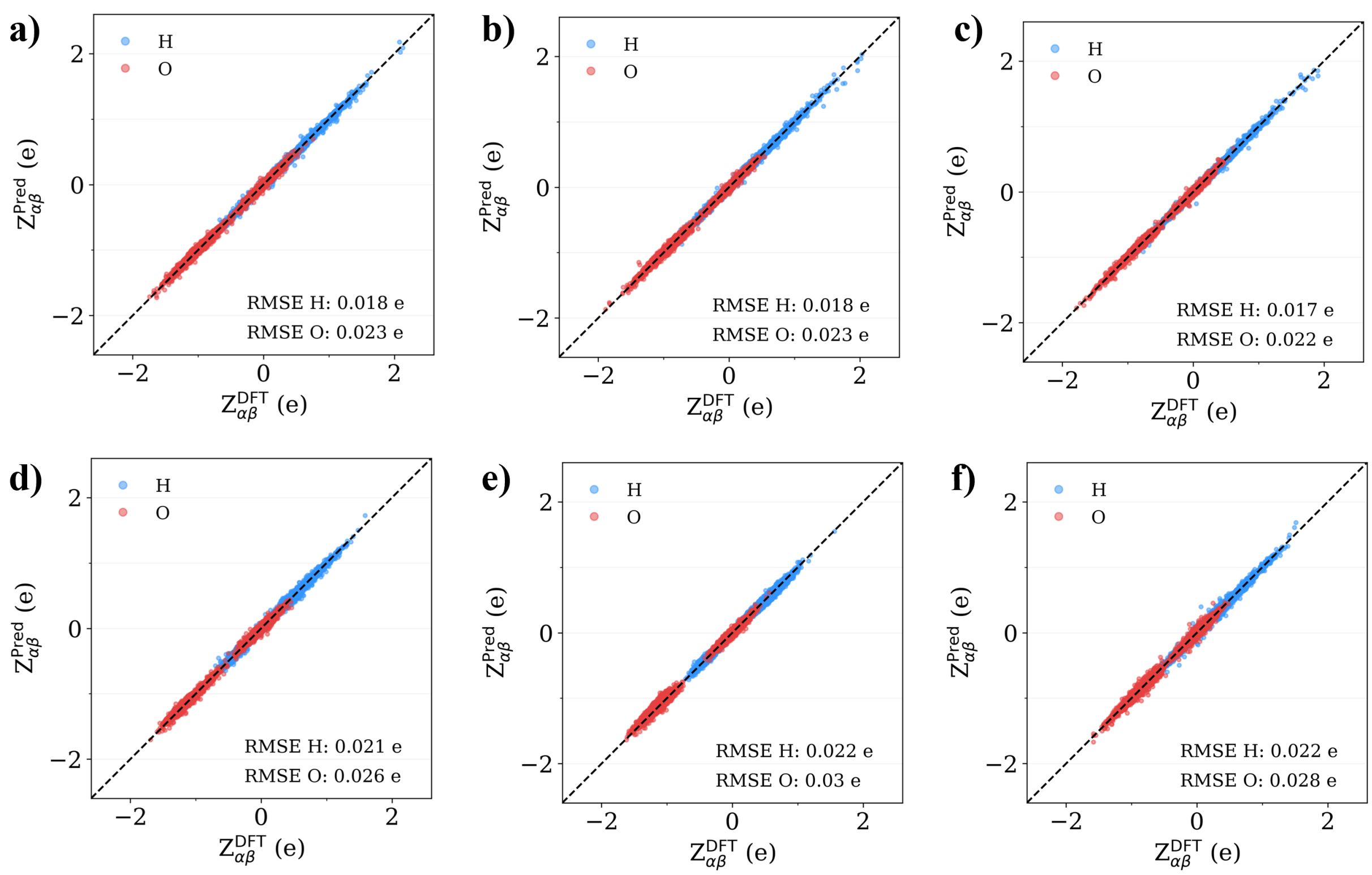

Figure S18: The prediction accuracy of BEC used for the electrostatic contribution to the forces in Figure S17 Panels a), b), and c) show data from the three trajectories "1a", "1b", and "3b" of protonated water at $E = 0.12$ V/Å, while panels d), e), and f) show data from the trajectories "1a", "2b", and "3a" of neat water at $E = 0.15$ V/Å.

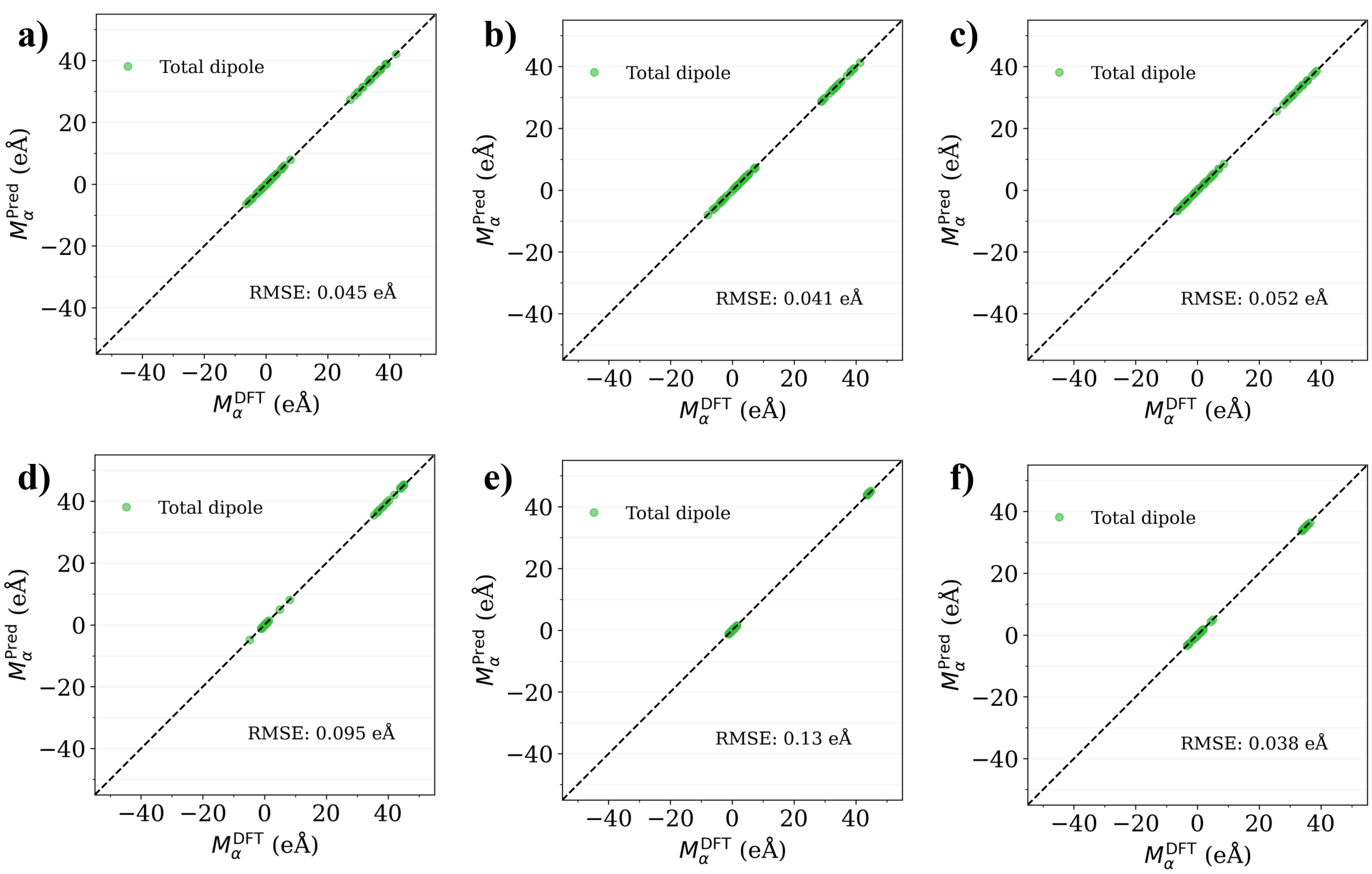

Figure S19: The accuracy of dipole predictions for the high-field geometries. Panels a), b), and c) show data from the three trajectories "1a", "1b", and "3b" of protonated water at $E =$ 0.12 V/Å, while panels d), e), and f) show data from the trajectories "1a", "2b", and "3a" of neat water at E = 0.15 V/Å.

13 Polarization convergence and relaxation in neat water

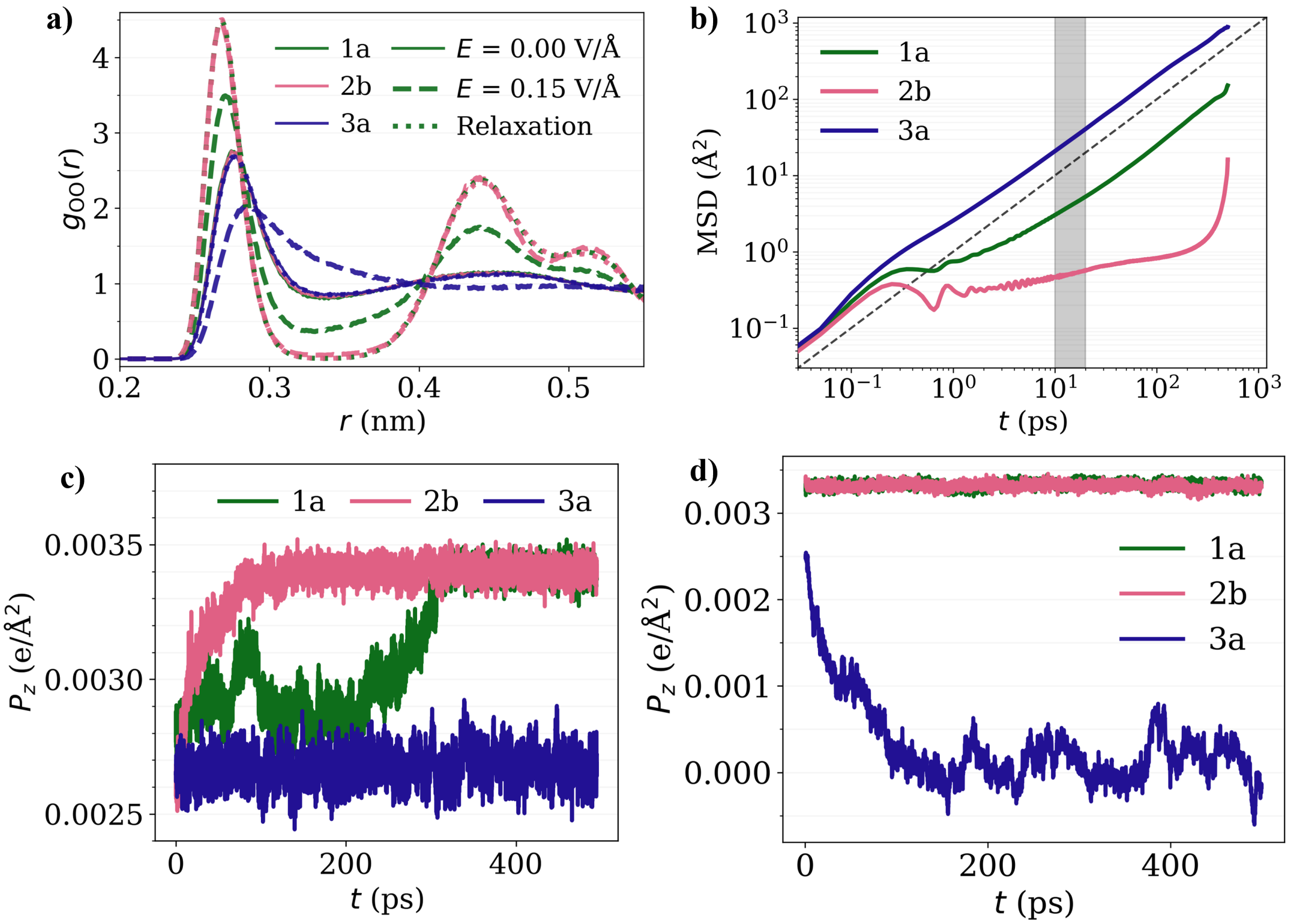

Figure S20: a) Oxygen-oxygen RDFs for trajectories 1a, 2b, and 3a of neat water at zero field, from step-function "experiments" ($E = 0.15$ V/Å switch-on) and from the relaxation "experiments" ($E = 0.15$ V/Å switch-off); b) The corresponding MSDs from the step-function experiments ($E = 0.15$ V/Å switch-on); c) The evolution of the total polarizations from the step-function "experiments" ($E = 0.15$ V/Å switch-on); d) The evolution of the total polarization from the relaxation "experiment" ($E = 0.15$ V/Å switch-off).

One visually clear structural change that neat water systems can undergo is the transition to an ice-like state (Figure S20a), where translational motion of individual water molecules is almost completely inhibited (Figure S20b). This transition is generally accompanied by a clear change in the system polarization. As a concrete example, we show the transient polarization during our independent trajectories of neat water at 0.15 V/Å in Figure S20c.

The trajectory "2b" quickly reaches a highly polarized and structured state (Figure S21a), while for the "1a" trajectory, this transition occurs after several hundred picoseconds (Figure S20c). Then, the "3a" trajectory (Figure S21b) becomes even less structured and more diffusive than the counterpart at zero field.

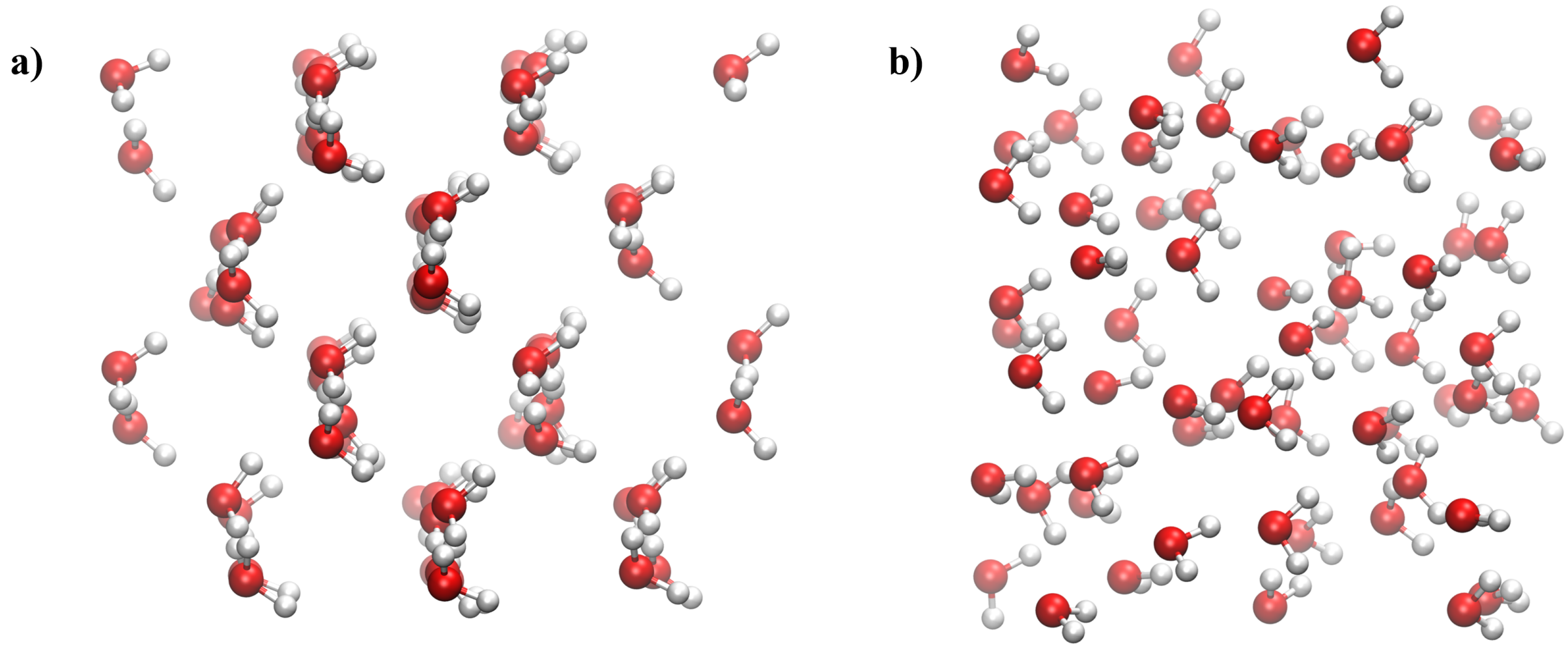

Figure S21: Representative snapshots of neat water simulated at $E = 0.15$ V/Å. The structure in panel a) is taken from trajectory "2b", and that in panel b) is taken from trajectory "3a". The dielectric constants extracted from the two simulations (Figure S20c) are 28 and 23, respectively. Additionally, if one computes the angle θ between the H-O-H angle bisector and the field direction (positive z-axis), the average is 13 degrees for trajectory "2b" and 22 degrees for trajectory "3a".

To further examine the properties of the neat water structures obtained at $E = 0.15$ V/Å, we performed the relaxation "experiments" where the field was turned off in the simulation and the evolution of the dipole moment and structure of the system was followed over time. The resulting polarization profiles are presented in Figure S20d. We can see that the two highly ordered "1a" and "1b" remained in the high polarization state for the full 500 ps even after the switch-off the applied E-field. The less ordered structure "3a", on the other hand, returned to the equilibrium water state after around 100 ps.

14 Structural analysis of H_3O^+(aq) in response to applied E-fields

One of the striking changes in the proton transfer reaction at high electric fields is an increased occurrence of (4a, 4b) coordination states. This increased likelihood of a hydrogen bond to the hydronium oxygen atom was also confirmed by the radial distribution function of this oxygen species (Figure S22b). For additional structural information, the solid angle formed by the three O-H vectors by placing the oxygen atom at the origin was computed across the different field strengths. On average, this angle is found to decrease at higher fields, hypothetically providing a donated hydrogen bond a more favorable interaction. The distribution of solid angles at 0.00, 0.04, and 0.15 V/Å is shown in Figure S22a.

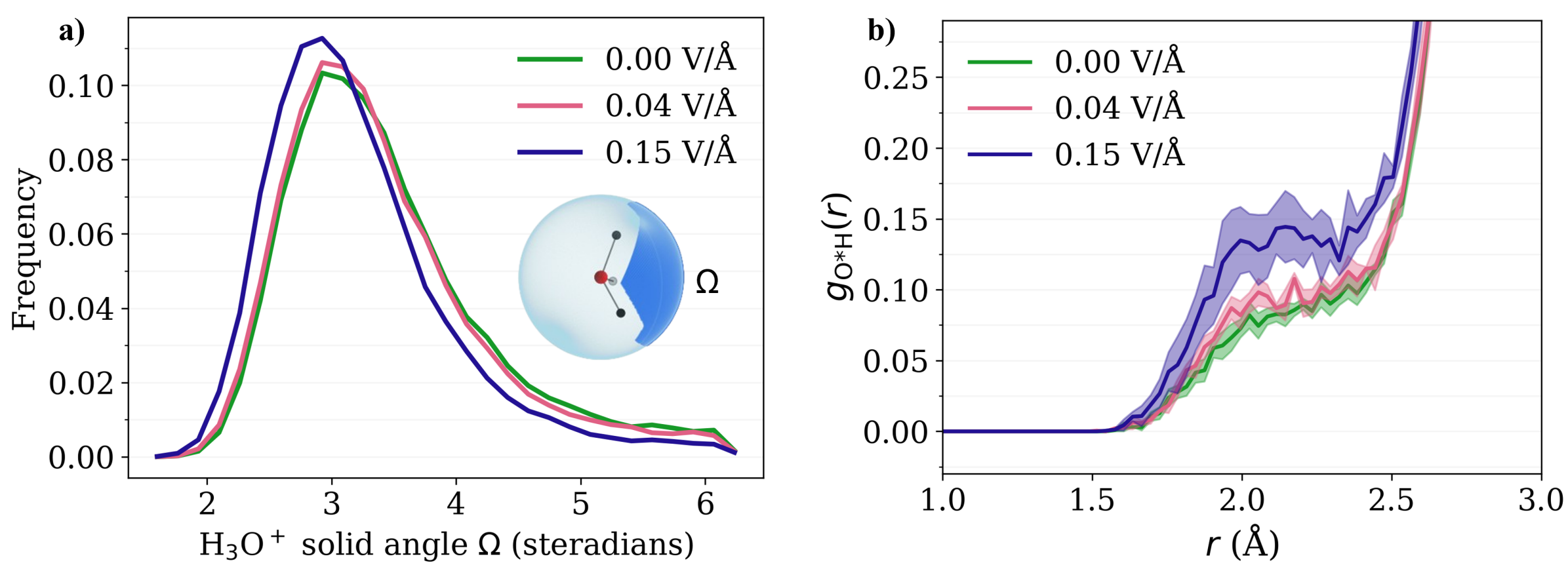

Figure S22: a) The solid angle formed by the three protons of a hydronium structure with respect to the central oxygen atom. For reference, a hemisphere has a solid angle of 2π steradians. b) The RDFs between hydrogen atoms H and the oxygen atom O^* of the instantaneous hydronium ion at selected field strengths. The shaded area corresponds to one standard deviation.

References

(S1) VandeVondele, J.; Krack, M.; Mohamed, F.; Parrinello, M.; Chassaing, T.; Hutter, J. Quickstep: Fast and accurate density functional calculations using a mixed Gaussian and plane waves approach. *Comput. Phys. Commun.* **2005**, *167*, 103–128.

(S2) Kühne, T. D.; Iannuzzi, M.; Del Ben, M.; Rybkin, V. V.; Seewald, P.; Stein, F.; Laino, T.; Khaliullin, R. Z.; Schütt, O.; Schiffmann, F.; Golze, D.; Wilhelm, J.; Chulkov, S.; Bani-Hashemian, M. H.; Weber, V.; Borštnik, U.; Taillefumier, M.; Jakobovits, A. S.; Lazzaro, A.; Pabst, H.; Müller, T.; Schade, R.; Guidon, M.; Andermatt, S.; Holmberg, N.; Schenter, G. K.; Hehn, A.; Bussy, A.; Belleflamme, F.; Tabacchi, G.; Glöß, A.; Lass, M.; Bethune, I.; Mundy, C. J.; Plessl, C.; Watkins, M.; VandeVondele, J.; Krack, M.; Hutter, J. CP2K: An Electronic Structure and Molecular Dynamics Software Package - Quickstep: Efficient and Accurate Electronic Structure Calculations. *J. Chem. Phys.* **2020**, *152*, 194103.

(S3) Furness, J. W.; Kaplan, A. D.; Ning, J.; Perdew, J. P.; Sun, J. Accurate and Numerically Efficient r2SCAN Meta-Generalized Gradient Approximation. *J. Phys. Chem. Lett.* **2020**, *11*, 8208–8215.

(S4) Goedecker, S.; Teter, M.; Hutter, J. Separable dual-space Gaussian pseudopotentials. *Phys. Rev. B* **1996**, *54*, 1703.

(S5) VandeVondele, J.; Hutter, J. Gaussian basis sets for accurate calculations on molecular systems in gas and condensed phases. *J. Chem. Phys.* **2007**, *127*, 114105.

(S6) Chen, M.; Ko, H.-Y.; Remsing, R. C.; Calegari Andrade, M. F.; Santra, B.; Sun, Z.; Selloni, A.; Car, R.; Klein, M. L.; Perdew, J. P.; others Ab initio theory and modeling of water. *Proc. Natl. Acad. Sci. U.S.A.* **2017**, *114*, 10846–10851.

(S7) Zeng, Z.; Wodaczek, F.; Liu, K.; Stein, F.; Hutter, J.; Chen, J.; Cheng, B. Mecha-

nistic insight on water dissociation on pristine low-index TiO2 surfaces from machine learning molecular dynamics simulations. *Nat. Commun.* **2023**, *14*, 6131.

(S8) Bussi, G.; Donadio, D.; Parrinello, M. Canonical sampling through velocity rescaling. *J. Chem. Phys.* **2007**, *126*, 014101.

(S9) Hjorth Larsen, A.; Jørgen Mortensen, J.; Blomqvist, J.; Castelli, I. E.; Christensen, R.; Dułak, M.; Friis, J.; Groves, M. N.; Hammer, B.; Hargus, C.; others The atomic simulation environmenta Python library for working with atoms. *J. Phys.: Condens. Matter* **2017**, *29*, 273002.

(S10) Liu, R.; Zhang, C.; Liang, X.; Liu, J.; Wu, X.; Chen, M. Structural and dynamic properties of solvated hydroxide and hydronium ions in water from ab initio modeling. *J. Chem. Phys.* **2022**, *157*, 024503.

(S11) Kuryla, D.; Berger, F.; Csányi, G.; Michaelides, A. How accurate are DFT forces? Unexpectedly large uncertainties in molecular datasets. *J. Chem. Phys.* **2025**, *163*, 224313.

(S12) Sudhama, A.; Zhang, Z.-Y.; Zhang, C. Foundation model distillation with PiNNAcLe: Application to the $ZnCl_2$ aqueous electrolyte solution. *Electrochim. Acta* **2026**, *572*, 149136.

(S13) Kingma, D. P.; Ba, J. Adam: A Method for Stochastic Optimization. 2017.

(S14) McDonnell, M. T.; Xu, H.; Keffer, D. J. Ab initio molecular dynamics simulations of an excess proton in a triethylene glycol–water solution: solvation structure, mechanism, and kinetics. *J. Phys. Chem. B* **2016**, *120*, 5223–5242.

(S15) de Buyl, P. tidynamics: A tiny package to compute the dynamics of stochastic and molecular simulations. *J. Open Source Softw.* **2018**, *3*, 877–877.

(S16) Umari, P.; Pasquarello, A. Ab initio Molecular Dynamics in a Finite Homogeneous Electric Field. *Phys. Rev. Lett.* **2002**, *89*, 157602.

(S17) Souza, I.; Íñiguez, J.; Vanderbilt, D. First-Principles Approach to Insulators in Finite Electric Fields. *Phys. Rev. Lett.* **2002**, *89*, 117602.

(S18) Ghosez, P.; Michenaud, J.-P.; Gonze, X. Dynamical atomic charges: The case of ABO3 compounds. *Phys. Rev. B* **1998**, *58*, 6224–6240.

(S19) Li, J.; Knijff, L.; Zhang, Z.-Y.; Andersson, L.; Zhang, C. PiNN: Equivariant Neural Network Suite for Modeling Electrochemical Systems. *J. Chem. Theory Comput.* **2025**, *21*, 1382–1395, PMID: 39883580.

(S20) Joll, K.; Schienbein, P.; Rosso, K. M.; Blumberger, J. Machine learning the electric field response of condensed phase systems using perturbed neural network potentials. *Nat. Comm.* **2024**, *15*, 8192.

(S21) Errington, J. R.; Debenedetti, P. G. Relationship between structural order and the anomalies of liquid water. *Nature* **2001**, *409*, 318–321.

(S22) Litman, Y.; Michaelides, A. Entropy Governs the Structure and Reactivity of Water Dissociation Under Electric Fields. *J. Am. Chem. Soc.* **2025**, *147*, 44885–44894.

(S23) Cassone, G.; Martelli, F. Electrofreezing of liquid water at ambient conditions. *Nat. Comm.* **2024**, *15*, 1856.
